# Pathway engineering strategies for improved product yield in yeast-based industrial ethanol production^☆^

**DOI:** 10.1016/j.synbio.2021.12.010

**Published:** 2022-01-22

**Authors:** Aafke C.A. van Aalst, Sophie C. de Valk, Walter M. van Gulik, Mickel L.A. Jansen, Jack T. Pronk, Robert Mans

**Affiliations:** a Department of Biotechnology, Delft University of Technology, Van der Maasweg 9, 2629, HZ Delft, the Netherlands; b DSM Biotechnology Centre, Alexander Fleminglaan 1, 2613, AX Delft, the Netherlands

**Keywords:** *Saccharomyces cerevisiae*, Synthetic biology, Metabolic engineering, Biofuels, Redox metabolism, Energy metabolism

## Abstract

Product yield on carbohydrate feedstocks is a key performance indicator for industrial ethanol production with the yeast *Saccharomyces cerevisiae*. This paper reviews pathway engineering strategies for improving ethanol yield on glucose and/or sucrose in anaerobic cultures of this yeast by altering the ratio of ethanol production, yeast growth and glycerol formation. Particular attention is paid to strategies aimed at altering energy coupling of alcoholic fermentation and to strategies for altering redox-cofactor coupling in carbon and nitrogen metabolism that aim to reduce or eliminate the role of glycerol formation in anaerobic redox metabolism. In addition to providing an overview of scientific advances we discuss context dependency, theoretical impact and potential for industrial application of different proposed and developed strategies.

## Graphical abstract

**Figure:**
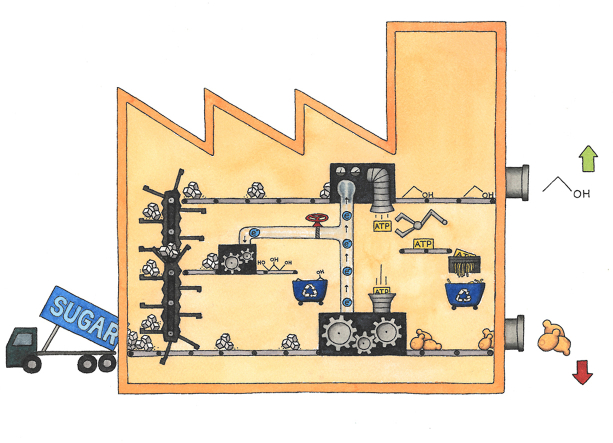

## 1 Introduction

In 2020, 99 billion liters of ethanol were produced by yeast-based fermentation of agriculture-derived carbohydrates [1]. Of this volume, approximately 30% was produced from Brazilian cane sugar (mainly consisting of sucrose) and approximately 54% from corn starch-derived glucose, mainly in the United States of America [1]. Ethanol is predominantly used as a renewable ‘drop-in’ transport fuel and ethanol-based value chains towards other compounds, including jet fuel and polyethylene, are under development [2,3].

Despite a plethora of academic and industrial studies on alternative microbial platforms [4], *Saccharomyces cerevisiae* remains the organism of choice for industrial ethanol production from carbohydrates. Factors that contribute to its popularity include rapid fermentation of glucose and sucrose to ethanol, insensitivity to phages, a long history of safe use in food applications and a high tolerance to ethanol. Ethanol concentrations in corn-starch-based, very-high-gravity fermentation processes can reach up to 21% (v/v) [5,6]. In bulk fermentation processes such as ethanol production, where costs of the carbohydrate feedstock can account for up to 70% of the total production costs [7], every detectable improvement of the ethanol yield on sugar is economically relevant. The extensive toolbox for genetic modification of *S. cerevisiae* [8] is therefore intensively used to explore options for maximizing ethanol yields by engineering its metabolic network.

**Fig. 1:**
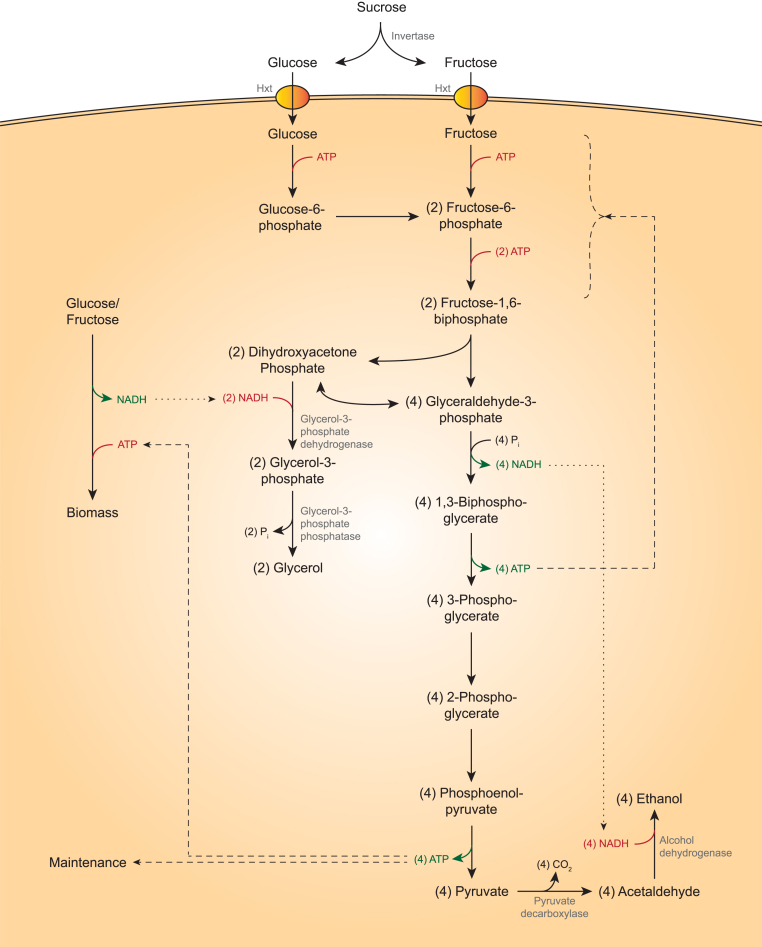
Schematic representation of the distribution of substrate over biomass, glycerol, ethanol and CO_2_ in anaerobically growing *S. cerevisiae*. NADH/NAD^+^ redox-cofactor coupling and use of ATP for sugar phosphorylation, biomass formation and maintenance are indicated by dotted and dashed arrows, respectively. Glucose, fructose and (after hydrolysis) sucrose are converted into pyruvate via the Emden-Meyerhoff glycolysis, yielding 2 NADH and 2 ATP per glucose equivalent. ATP is used for cellular maintenance and synthesis of biomass (growth). NADH is primarily re-oxidized via alcoholic fermentation, but a surplus of NADH formed during biomass synthesis is re-oxidized via the production of glycerol.

In *S. cerevisiae*, anaerobic metabolism of glucose or sucrose starts with their conversion to pyruvate via the ATP-generating Embden-Meyerhof glycolytic pathway. NADH generated by this oxidative pathway is re-oxidized by the combined action of pyruvate decarboxylase (Pdc1, Pdc5, Pdc6, EC 4.1.1.1: pyruvate → acetaldehyde + CO_2_) and NAD^+^-dependent alcohol dehydrogenases (predominantly Adh1, EC 1.1.1.1: acetaldehyde + NADH → ethanol + NAD^+^ [9]) (Fig. 1). This native yeast pathway for alcoholic fermentation perfectly conserves the degree of reduction of sugars [10] and almost completely captures their heat of combustion in ethanol (−2840 kJ per mol of glucose versus −2734 kJ per two mol of ethanol). Clearly, if alcoholic fermentation was the only relevant metabolic process in industrial ethanol production, attempts to improve ethanol yields on sugars as sole carbon and electron sources would be futile. Metabolic engineering strategies for improving ethanol yields are therefore directly or indirectly related to another cellular process that occurs during industrial ethanol production: anaerobic growth.

In the absence of growth, survival of yeast cells requires cellular maintenance metabolism, which encompasses use of ATP for growth-independent processes that maintain structural integrity and viability [11]. In anaerobic yeast cultures, this ATP is exclusively generated via alcoholic fermentation (Fig. 1). In contrast, growth of yeast cells not only requires ATP but also organic precursors for biomass components, whose biosynthetic pathways compete for carbon with ethanol production (Fig. 1). Anaerobic growth occurs in all current industrial processes for ethanol production and the resulting surplus yeast biomass is valorized by its inclusion in a by-product stream sold as an animal feed supplement [12].

Growth is coupled to formation of glycerol, a second important byproduct of anaerobic yeast metabolism, by redox-cofactor metabolism. Formation of *S. cerevisiae* biomass from sugar, ammonium or urea and other nutrients is coupled to a net reduction of NAD^+^ to NADH [13,14] (Fig. 1). Anaerobic *S. cerevisiae* cultures cannot re-oxidize this NADH by mitochondrial respiration and instead rely on NADH-dependent reduction of the glycolytic intermediate dihydroxyacetone-phosphate to glycerol-3-phosphate, in a reaction catalysed by NAD^+^-dependent glycerol-3-phosphate dehydrogenase (Gpd1, Gpd2, EC 1.1.1.8 [15,16]). Glycerol-3-phosphate is then hydrolyzed by glycerol-3-phosphate-phosphatase (Gpp1, Gpp2, EC 3.1.3.21 [17]) to yield phosphate and glycerol (Fig. 1). In processes based on wild-type *S. cerevisiae* strains, approximately 4% of the potential ethanol yield on carbohydrate feedstocks was estimated to be lost to glycerol [18]. Based on current ethanol production volumes, this loss would correspond to approximately 4 billion liters of ethanol per year.

The aim of this paper is to review the current body of knowledge on pathway engineering strategies that focus on maximizing ethanol yields on glucose or sucrose by altering the ratio of ethanol, biomass and glycerol formation in *S. cerevisiae*. This scope excludes a large body of metabolic engineering research aimed at expanding the sugar- and polysaccharide substrate range of *S. cerevisiae* to enable its nascent application for industrial-scale fermentation of lignocellulosic hydrolysates generated from agricultural residues or energy crops (reviewed in Refs. [4,[19], [20], [21]]). However, the discussed strategies can, in principle, be applied in such ‘second-generation’ bioethanol processes as well as in ‘first-generation’ processes based on corn starch or cane sugar, once other metabolic engineering strategies have been successfully addressed.

## 2 Process conditions

Growth of anaerobic laboratory cultures of wild-type *S. cerevisiae* strains under different conditions provided insight in how distribution of sugar over biomass, glycerol and ethanol can be influenced and have therefore been a key source of inspiration for the design of metabolic engineering strategies.

**Fig. 2:**
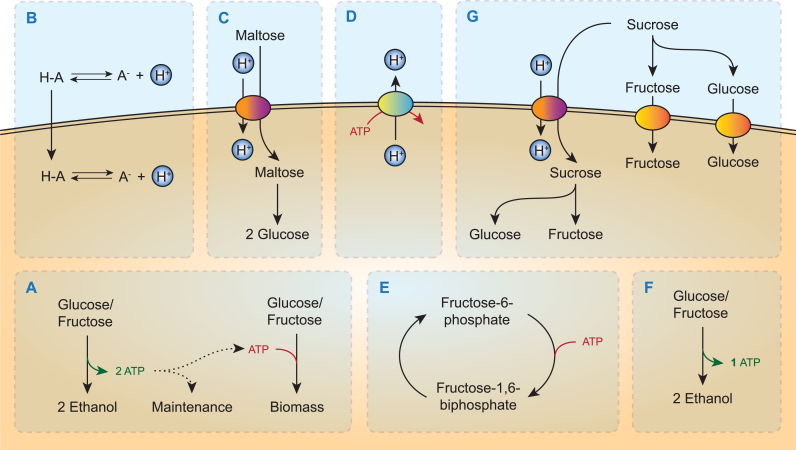
Schematic representation of energy metabolism in *S. cerevisiae* and strategies to improve ethanol yield on sugar. A: Alcoholic fermentation of glucose or fructose. B: Maintenance energy requirements can be increased by presence of weak organic acids in culture medium. C: The net ATP yield (mol ATP/mol glucose equivalent) of maltose utilization is lower than that of glucose, since maltose transport is proton coupled, whereas glucose is transported via facilitated diffusion. D: Plasma membrane ATPase exports protons at the cost of ATP. E: Example of a futile cycle, e.g. a set of reactions that leads to net hydrolysis of ATP, that can be introduced in order to enforce ‘ATP wasting’. F: The Enter-Doudoroff glycolytic pathway yields only 1 ATP per glucose equivalent, instead of 2 ATP. G: Intracellular targeting of invertase (iSuc2) combined with uptake of sucrose by proton symport (left) lowers the ATP yield compared to wildtype *S. cerevisiae*, where sucrose is hydrolyzed extracellularly, after which the resulting monosaccharides are taken up via facilitated diffusion (right).

In anaerobic, sugar-limited cultures of *S. cerevisiae*, maintenance-energy requirements are essentially growth-rate independent [[22], [23], [24]]. The fraction of the consumed sugar that is fermented to ethanol therefore increases with decreasing specific growth rate [11] (Fig. 2A). This correlation is clearly demonstrated in anaerobic retentostat cultures of *S. cerevisiae*, in which all biomass is retained in the culture and only cell-free effluent leaves the reactor. In such systems, near-theoretical ethanol yields on glucose were demonstrated during prolonged growth at near-zero specific growth rates [22].

As an alternative to reducing the specific growth rate, the fraction of the sugar substrate that is fermented to ethanol by actively growing anaerobic cultures to meet maintenance-energy requirements can be increased by changing cultivation conditions. In particular, addition of weak organic acids such as lactate, acetate, propionate or benzoate to anaerobic batch and chemostat cultures grown at low pH, was shown to lead to lower biomass yields and higher ethanol yields [13,[25], [26], [27], [28], [29]]. These results reflect an increased maintenance energy requirement for intracellular pH homeostasis, caused by an influx of protons into the yeast cytosol as a result of weak acid diffusion (Fig. 2B) [30]. In anaerobic yeast cultures, countering this ‘weak acid uncoupling’ and maintenance of intracellular pH homeostasis critically depends on ATP-dependent proton export by the plasma membrane ATPase (Pma1, EC 7.1.2.1) (Fig. 2D) [31,32]. These observations clearly indicate the potential of modifying maintenance-energy requirements as a means to improve ethanol yields. Practical issues such as costs of adding organic acids and their subsequent removal from process effluents, as well as potential synergies of weak organic acid and ethanol toxicity [33,34], preclude direct application of weak organic acid uncoupling in industrial bioethanol production. Feedstocks for second-generation bioethanol production already contain inhibitors such as acetic acid, furfural and hydroxymethyl-2-furaldehyde [21,[35], [36], [37]], which cause increased ATP requirements for cellular maintenance. In addition, high concentrations of ethanol also in themselves affect maintenance energy requirements by increasing permeability of the yeast plasma membrane to protons and thereby activating Pma1 [38,39].

Experiments on disaccharide metabolism by anaerobic *S. cerevisiae* cultures provided a first demonstration that ethanol yields can be modified by changing the mechanism of sugar import. In contrast to transport of glucose, which occurs via facilitated diffusion by Hxt transporters [40,41], uptake of its dimer maltose by *S. cerevisiae* is mediated by Malx1 transporters and involves symport with a single proton [32,42]. After intracellular hydrolysis of maltose by a Malx2 maltase (EC 3.2.1.20, maltose + H_2_O → 2 glucose), alcoholic fermentation of the resulting two glucose molecules yields 4 molecules of ATP. However, since one of these ATP molecules has to be used to enable expulsion of the symported proton by Pma1, which has a stoichiometry of 1H^+^/ATP [32,42], the net ATP yield from maltose fermentation is only 1.5 ATP per glucose equivalent (Fig. 2C). Indeed, based on hexose units, ethanol and biomass yields of *S. cerevisiae* in anaerobic maltose-limited chemostat cultures were shown to be 16% higher and 25% lower, respectively, than in corresponding glucose-limited cultures [31]. These observations inspired metabolic engineering studies that were focused on sucrose-containing feedstock for bioethanol production.

During growth on ammonium or urea [43,44], a significant part of the ‘surplus’ NADH generated in biosynthesis is derived from the synthesis of amino acids from these nitrogen sources and sugar. Several studies reported lower glycerol yields and higher ethanol yields on sugar in anaerobic cultures grown with amino acids or yeast extract as the nitrogen source [[45], [46], [47]]. Although use of amino acids as industrial nitrogen source is not an economically viable proposition, these observations highlighted the potential for engineering redox-cofactor metabolism to improve ethanol yields.

## 3 Engineering of energy coupling

### 3.1 Introduction of futile cycles

Several metabolic engineering strategies have been explored to increase the use of sugar for cellular maintenance energy requirements by introducing metabolic ‘futile cycles’, whose net effect is the hydrolysis of ATP to ADP and inorganic phosphate with a concomitant release of heat (Fig. 2E). Such ‘ATP wasting’ cycles can either be introduced by constitutive expression of ATPases or by creating more complicated futile cycles that cause a net hydrolysis of ATP. Overexpression of the soluble F1 unit of the *Escherichia* *coli* H^+^-ATPase in *S. cerevisiae* [48,49] led to a 10% increase of the anaerobic ethanol yield on glucose relative to a reference strain, but also caused a 26% decrease of the specific growth rate [48]. Overexpression of *PHO5* or *PHO8*, which encode aspecific phosphatases (EC 3.1.3.1/2) [50,51] was similarly reported to cause increased ATP turn-over. *PHO8* overexpression was reported to cause a 17% higher ethanol yield on glucose, without affecting growth rate [50]. Simultaneous activity of ATP-generating glycolytic and ATP-consuming gluconeogenic enzymes leads to textbook examples of futile metabolic cycles. Though not tested with the specific aim to improve ethanol yields, overexpression of the gluconeogenic enzyme fructose-1,6-bisphosphatase (Fbp1, EC 3.1.3.11: fructose-1,6-bisphosphate + H_2_O → fructose-6-phosphate + P_i_) increased glucose consumption (19%) and CO_2_ (10%) and ethanol (14%) production rates of aerobic suspensions of non-growing cells [52]. An even more pronounced effect on the ethanol production rate (22%) was found when the gluconeogenic enzyme phosphoenolpyruvate carboxykinase (PEPCK, EC 4.1.1.49: oxaloacetate + ATP → phosphoenolpyruvate + ADP + CO_2_) was simultaneously overexpressed [52,53]. More recently, *E. coli* PEPCK (*pckA*) was overexpressed together with the yeast anaplerotic enzyme pyruvate carboxylase (Pyc2, EC 6.4.1.1: pyruvate + ATP + CO_2_ → oxaloacetate + ADP + Pi) [54]. Simultaneous activity of these enzymes results in hydrolysis of two ATP molecules for the formation of phosphoenolpyruvate (PEP) from pyruvate. Since, in glucose-grown cultures, the glycolytic enzyme pyruvate kinase (Pyk2, Cdc19, EC 2.7.1.40) converts PEP back to pyruvate with the formation of only a single ATP, the net result of this futile cycle is the hydrolysis of one ATP. The potential of this strategy was demonstrated by more ethanol production, related to yeast biomass, by the overexpression strain than by the control strain [54].

An inherent risk of the constitutive expression of futile cycles is that, in industrial processes, situations may occur in which a too large drain of the cellular ATP content can no longer be compensated for by faster alcoholic fermentation. In extreme situations, net ATP synthesis might even decrease below maintenance energy-requirements and cause cell death. Careful ‘tuning’ of the *in vivo* activity of engineered futile cycles can, in principle, address this problem in cultures grown under constant conditions in the laboratory. However, such tuning would be much more difficult to achieve in large-scale industrial processes, which are highly dynamic, for example as a consequence of changing sugar and ethanol concentrations. Application-oriented pathway-engineering studies therefore mostly focus on strategies that, instead, aim at a fixed, stoichiometric reduction of the ATP yield from ethanol fermentation.

### 3.2 Decreasing the ATP stoichiometry of yeast glycolysis

The bacterium *Zymomonas mobilis* employs the Entner-Doudoroff (ED) pathway for alcoholic fermentation. Instead of the 2 mol ATP/mol glucose generated in yeast glycolysis, this pathway has a net ATP yield of only 1 mol ATP/mol glucose [55,56]. As a consequence, high ethanol yields can be achieved in growing *Z. mobilis* cultures [55,56]. A now expired patent proposed functional expression of the ED pathway in *S. cerevisiae* (Fig. 2F) [57]. However, experimental studies failed to achieve the high *in vivo* activities of 6-phosphogluconate dehydratase (PGDH, EC 4.2.1.12: 6-phosphogluconate → 2-dehydro-3-deoxy-gluconate-6-phosphate) in *S. cerevisiae* that would be required to demonstrate an impact on ethanol yield [58,59]. A limiting activity of PGDH, which contains an [4Fe-4S] iron-sulfur cluster [60], was attributed to the well-documented difficulties in expressing heterologous iron-sulfur-cluster enzymes in the yeast cytosol [61].

An alternative approach to reduce the ATP yield of glycolysis in *S. cerevisiae* was based on functional expression of a heterologous, non-phosphorylating, NADP^+^-dependent glyceraldehyde-3-phosphate dehydrogenase (GAPN, EC 1.2.1.9: glyceraldehyde-3-phosphate + NADP^+^ → 3-phosphoglycerate + NADPH), which bypasses the ATP-generating phosphoglycerate kinase reaction (Pgk1, EC 2.7.2.3: 1,3-biphosphoglycerate + ADP → 3-phosphoglycerate + ATP) [[62], [63], [64]]. Strains engineered with this strategy increased the ethanol yield in anaerobic cultures by 3% [64] and 7.6% [62]. This increase was partly attributed to a lower ATP yield of glycolysis and partly to changes in redox-cofactor metabolism (see 4.2).

### 3.3 Altering topology and energy coupling of disaccharide metabolism and transport

In contrast to maltose which, as described above, is taken up by proton symport prior to hydrolysis [31,32,42], sucrose metabolism in wild-type *S. cerevisiae* strains is predominantly initiated by its extracellular hydrolysis to glucose and fructose, catalysed by invertase (Suc2, EC 3.2.1.26) (Fig. 1) [65,66]. After uptake via facilitated diffusion, mediated by Hxt transporters [67], these hexoses are oxidized to pyruvate by yeast glycolysis.

Due to the presence of a second start codon in the *SUC2* transcript, a small fraction of the expressed invertase is retained in the cytosol [66] while, moreover, the Mal11 (Agt1) maltose-proton symporter is also able to import sucrose [68,69]. Replacement of the native *SUC2* gene by a constitutively expressed, truncated *SUC2* gene that no longer encoded the N-terminal excretion sequence of Suc2 led to a near-complete targeting of invertase to the yeast cytosol (Fig. 2G) [70]. Adaptive laboratory evolution of an engineered *S. cerevisiae* strain expressing this internal invertase (‘iSuc2’) in anaerobic, sucrose-limited chemostat cultures yielded an evolved strain with increased expression of *MAL11*. When compared under identical conditions in anaerobic chemostat cultures, the evolved strain showed an 11% higher ethanol yield and a 30% lower biomass yield on sucrose than the reference strain [70]. These results were in good agreement with predictions based on stoichiometric models of yeast metabolism and mirrored earlier comparisons of biomass and product yields of wild-type *S. cerevisiae* grown anaerobically on maltose and glucose [31]. Using a similar strategy, it should also be possible to decrease the ATP yield of monosaccharide dissimilation by replacing the endogenous facilitated diffusion transporters by proton symporters [71,72].

## 4 Engineering of redox metabolism

Multiple pathway engineering strategies for improving ethanol yield on sugars aim to minimize production of glycerol. In aerobic *S. cerevisiae* cultures, generation of glycerol-3-phosphate by the Gpd1 and Gpd2 glycerol-3-phosphate dehydrogenases is non-essential due to the presence of an alternative route for glycerolipid synthesis that involves 1-acyldihydroxyacetone-phosphate as intermediate [73]. In contrast, due to the essential role of glycerol formation in NADH redox-cofactor balancing in non-respiratory cultures, double deletion of *GPD1* and *GPD2* prevents anaerobic growth [74,75]. Anaerobic growth of *gpd1Δ gpd2Δ* strains can be rescued by supplementation of compounds such as acetaldehyde or acetoin, which can be reduced by intracellular NADH-dependent dehydrogenases [74,75]. Glycerol-negative mutants are highly sensitive to osmotic stress due to the key role of glycerol in osmotolerance of *S. cerevisiae* [74,76].

**Fig. 3:**
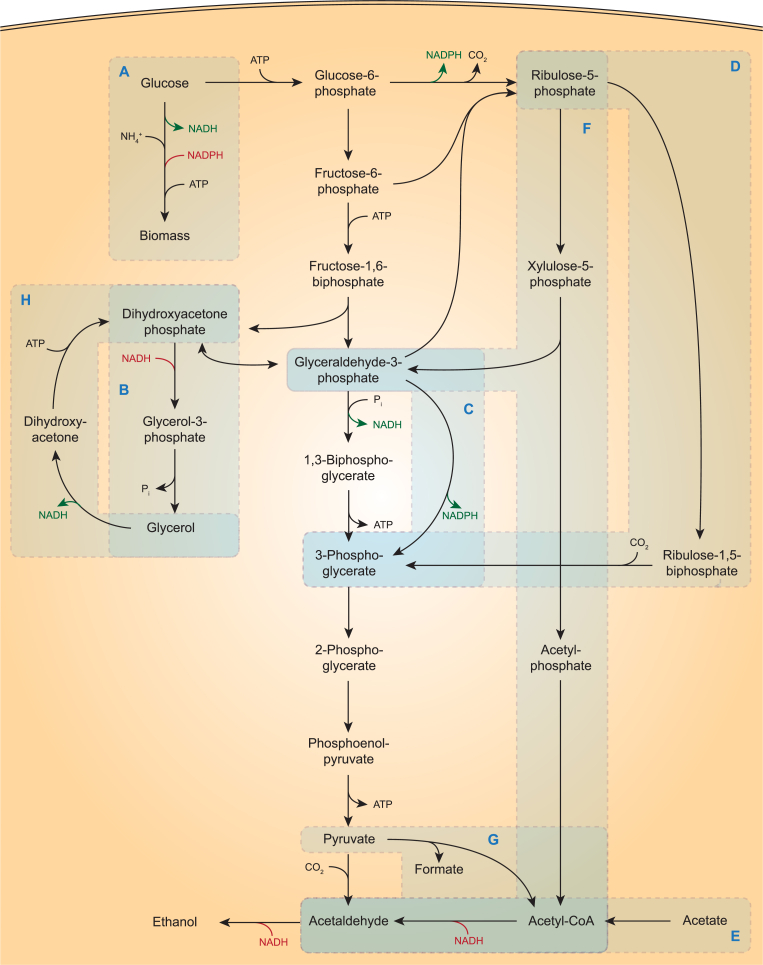
Schematic representation of pathway engineering strategies for minimizing formation of glycerol as ‘redox’ sink for re-oxidation of NADH generated in biosynthetic reactions during anaerobic growth of *S. cerevisiae*. A: Biosynthetic reactions require a net input of ATP and NADPH, while yielding NADH. Ammonium assimilation is the key contributor to NADH production, and replacing the NADP^+^-dependent step by an NADH-dependent step, can reduce the NADH production in biosynthetic reactions. B: Native glycerol pathway. C: Bypass of NAD^+^-dependent glyceraldehyde-3-phosphate dehydrogenase by heterologous non-phosphorylating, NADP^+^-dependent glyceraldehyde-3-phosphate dehydrogenase (GAPN). D: Non-oxidative bypass of NAD^+^-dependent glyceraldehyde-3-phosphate dehydrogenase by heterologously expressed phosphoribulokinase and ribulose-1,5-bisphosphate carboxylase/oxygenase (Rubisco). E: Re-oxidation of NADH by A-ALD-expressing strain, using exogenous acetate as electron acceptor. F: Re-oxidation of NADH enabled by combined expression of heterologously expressed NADH-dependent acetylating acetaldehyde dehydrogenase (A-ALD), phosphoketolase and phosphotransacetylase. G: Re-oxidation of NADH enabled by combined expression of heterologously expressed A-ALD and pyruvate-formate lyase. H: Combined expression of a heterologous NADH-dependent glycerol dehydrogenase and the native dihydroxyacetone kinase enables ethanol formation from glycerol when combined with strategies D, E, F and/or G.

In anaerobic, glucose-limited cultures of *S. cerevisiae* grown on synthetic media with ammonium as nitrogen source, approximately 12 mmol of glycerol is formed per gram of biomass dry weight [74,77], which closely matches calculated requirements for NADH re-oxidation [78]. Strain-dependent diversity in glycerol production may reflect different biomass composition, formation of metabolites whose formation is coupled to a net generation of NADH (e.g. acetate [75]) and/or activity of the γ-butyric acid (GABA) shunt [79]. ‘Tuning’ of *in vivo* activities of glycerol-3-phosphate dehydrogenase, by deletion of either *GPD1* or *GPD2* or by promoter engineering, has in different wild-type *S. cerevisiae* strain backgrounds and under different (semi-) anaerobic cultivation conditions, been shown to affect specific growth rates, glycerol and ethanol yields (Fig. 3B) [18,74,75,80]. While biomass synthesis in *S. cerevisiae* results in a net reduction of NAD ^+^ to NADH, it requires a net oxidation of NADPH to NADP^+^ [81,82]. Based on this observation, Anderlund et al. (1999) [83] and Nissen et al. (2001) [84] explored whether expression of heterologous soluble transhydrogenases (EC 1.6.1.1: NADPH + NAD^+^ → NADP^+^ + NADH) from *E. coli* or *Azotobacter vinelandii*, respectively, could convert the ‘surplus’ NADH generated by anaerobic *S. cerevisiae* cultures into NADPH and thereby lower glycerol production. Physiological analysis of the resulting strains revealed that, instead, intracellular concentrations of reduced and oxidized forms of these cofactors favoured the reverse reaction, thus resulting in higher glycerol yields and a lower ethanol yields than in the corresponding reference strains [83,84].

### 4.1 Engineering redox-cofactor coupling of nitrogen assimilation

Based on observations that amino acid synthesis from ammonium or urea is a key contributor to the ‘excess’ NADH formed in yeast biosynthesis, an early redox engineering study [18] focused on Gdh1, the NADP^+^-dependent glutamate dehydrogenase (EC 1.4.1.4) that catalyses the key reaction in ammonium assimilation by nitrogen-sufficient *S. cerevisiae* cultures: (2-oxoglutarate + NH_4_^+^ + NADPH → glutamate + NADP^+^, Fig. 3A). Theoretical analysis predicted that making ammonium assimilation NADH-dependent could reduce glycerol production in anaerobic cultures by half. In one strategy, deletion of *GDH1* was combined with constitutive overexpression of *GLN1* and *GLT1*, which encode ATP-dependent glutamine synthetase (GS, EC 6.3.1.2: glutamate + NH_4_^+^ + ATP → glutamine + ADP + P_i_) and NADH-dependent glutamate-2-oxoglutarate aminotransferase (EC 1.4.1.14: glutamine + 2-oxoglutarate + NADH + H^+^ → 2 glutamate + NAD^+^ (GOGAT), respectively. In anaerobic bioreactor batch cultures, a resulting engineered strain grew at 90% of the specific growth rate of the reference strain, while its glycerol yield on glucose was 38% lower and its ethanol yield was 10% higher [43]. The increased ethanol yield was attributed to a combination of reduced NADH formation and increased ATP consumption in ammonium assimilation. In a second strategy, deletion of *GDH1* was combined with overexpression of the NADH-dependent glutamate dehydrogenase *GDH2* (EC 1.4.1.2: 2-oxoglutarate + NH_4_^+^ + NADH → glutamate + NAD^+^). This approach led to a 30% lower glycerol yield. However, the ethanol yield was hardly affected and the biomass yield was 12% higher than that of the reference strain. This observation was attributed to a reduced loss of carbon via CO_2_ formation in the oxidative pentose-phosphate pathway [18], which is the main source of NADPH in *S. cerevisiae* [85,86]. Since NADH re-oxidation in the first step of ammonium assimilation cannot completely replace glycerol formation, the GS-GOGAT strategy, as successfully implemented by Nissen et al. (2000) [43], left room for further reduction of glycerol yields.

### 4.2 Expression of NADP^+^-dependent, non-phosphorylating glyceraldehyde 3-phosphate dehydrogenase (gapN)

In *S. cerevisiae*, the oxidative step in glycolysis is catalysed by the strictly NAD^+^-dependent oxidation of glyceraldehyde-3-phosphate to 1,3-diphosphoglycerate by isoenzymes of glyceraldehyde-3-phosphate dehydrogenase (Tdh1, 2 or 3, EC 1.2.1.12). Based on stoichiometric modelling of yeast metabolism, Bro et al. (2006) identified expression of a heterologous non-phosphorylating, NADP^+^-dependent glyceraldehyde-3-phosphate dehydrogenase (GAPN), which generates 3-phosphoglycerate instead of 1,3-diphosphoglycerate, as a promising option to increase ethanol yields (Fig. 3C). Initial experimental verification of this model prediction by expression of *Streptococcus mutans gapN* showed a 44% lower glycerol yield in anaerobic, glucose-grown batch cultures than in a reference strain. No negative impact on specific growth rate or biomass yield was observed, but also the ethanol yield on glucose was not significantly altered [64]. Subsequent studies in which expression of *Bacillus cereus gapN* was tested, reported a 3.5% higher final ethanol concentration and a 23% reduction of the glycerol yield on sugar relative to a reference strain [63]. Expression of *Bacillus cereus gapN* in combination with deletion of *GPD1*, yielded a strain that exhibited a 49% lower glycerol yield and 8% higher ethanol yield than the wild-type reference strain. However, the engineered strain was found to be highly sensitive to osmotic stress, thereby precluding its use in high-gravity industrial ethanol fermentation. When osmotolerance was restored by overexpression of *TPS1* and *TPS2*, which encode trehalose-6-phosphate synthase (EC 2.4.1.15: glucose-6-phosphate + UDP-glucose → UDP + trehalose-6-phosphate) and trehalose-6-phosphate phosphatase (EC 3.1.3.12: trehalose-6-phosphate + H_2_O → trehalose + P_i_), near-wild-type anaerobic growth rates were reported along with an up to 8% higher ethanol yield and 73% lower glycerol yield, respectively [62]. In a further study [87], expression of *gapN* from *Streptococcus mutans* was combined with deletion of *FPS1*, which encodes a membrane channel protein involved in glycerol export, in some strains combined with overexpression of *UTR1*, which encodes *S. cerevisiae* NADH kinase (EC 2.7.1.86: ATP + NADH → ADP + P_i_ + NADPH) [88]. While lower glycerol yields and higher ethanol yields were observed in micro-aerobic cultures, the engineered strains were unable to grow under fully anaerobic conditions.

### 4.3 NADH-dependent reduction of acetate to ethanol

In many fermentative bacteria, acetylating acetaldehyde dehydrogenase (A-ALD, EC1.2.1.10: acetyl-CoA + NADH + H^+^ → acetaldehyde + CoA + NAD^+^) catalyses a key reaction in alcoholic fermentation, that is followed by NADH-dependent reduction of acetaldehyde to ethanol [89]. The potential of using the combination of A-ALD and yeast alcohol dehydrogenase to re-oxidize NADH in anaerobic *S. cerevisiae* cultures, and thereby replace glycerol as NADH redox sink for ethanol, was explored by expressing the A-ALD-encoding *E. coli* gene *mhpF* in a *gpd1Δ gpd2Δ* strain [90]. Like other *gpd1Δ gpd2Δ S. cerevisiae* strains, the resulting strain did not grow anaerobically on glucose as sole carbon source. However, anaerobic growth was restored by addition of acetate to growth media (Fig. 3E). In anaerobic *S. cerevisiae* cultures, acetate is activated to acetyl-CoA the acetyl-CoA synthethase isoenzyme Acs2 (EC 6.2.1.1: acetate + ATP + CoA → acetyl-CoA + AMP + PP_i_, [91]). In anaerobic bioreactor batch cultures supplemented with 2 g/L acetate, the engineered strain did not produce glycerol and showed a 13% higher apparent ethanol yield on glucose (note that part of the produced ethanol was derived from acetate rather than from glucose). Under these conditions, the *mhpF*-expressing strain grew at 44% of the specific growth rate of the *GPD1 GPD2* reference strain [90]. Introduction, in the same *gpd1Δ gpd2Δ* genetic background, of a single copy of an expression cassette for *eutE*, an alternative *E. coli* A-ALD gene, increased specific growth rate to 84% of that of the reference strain [92].

When *E. coli* EutE was expressed in a *GPD1 GPD2 S. cerevisiae* strain, a mere 10% reduction of the amount of glycerol produced per gram biomass was observed in anaerobic, glucose-grown batch cultures supplemented with acetate. This observation indicated that the native glycerol pathway effectively competed with *E. coli* EutE for NADH in this genetic context. Deletion of *GPD2*, which encodes the redox-regulated isoenzyme of glycerol-3-phosphate dehydrogenase in *S. cerevisiae*, led to an 80% reduction of glycerol production, with a corresponding increase in acetate consumption [92].

Acetate is a common constituent and inhibitor of yeast performance in the hydrolysates of lignocellulosic biomass that are explored as feedstocks for ‘second-generation’ yeast-based ethanol production [93]. Since, in such processes, expression of A-ALD offers an option to convert an inhibitor into additional product, further pathway engineering strategies were explored to increase the amount of NADH available for acetate reduction and to improve robustness of engineered *gpd1Δ gpd2Δ*, A-ALD-expressing strains. To enable additional NADH generation, the native *S. cerevisiae* NADP^+^-dependent 6-phosphogluconate dehydrogenases Gnd1 and Gnd2 (EC 1.1.1.44: 6-phosphogluconate + NADP^+^ → ribulose-5-phosphate + CO_2_ + NADPH) were replaced by the NAD^+^-dependent enzyme GndA from *Methylobacillus flagellates* (EC 1.1.1.343). To force flux through the resulting, now partially NADH-coupled oxidative pentose-phosphate pathway, *ALD6*, which encodes NADP^+^-dependent acetaldehyde dehydrogenase (EC 1.2.1.5: acetaldehyde + NADP^+^ → acetate + NADPH), was deleted. This metabolic engineering strategy resulted in a 29% higher acetate consumption per gram biomass than in the parental *gpd1Δ gpd2Δ*, EutE-expressing strain [94]. Relative to a congenic *GPD1 GPD2* reference strain, the engineered strain showed a 13% higher ethanol yield and a 29% lower specific growth rate.

An alternative strategy to boost the acetate-reducing capacity of EutE-expressing strains focused on changing the cofactor preference of alcohol dehydrogenase, which in *S. cerevisiae* is strictly NADH-dependent [95]. Relative to an industrial *S. cerevisiae* strain expressing *Bifidobacterium adolescentis* EutE in a *gpd1Δ gpd2Δ* background, a further engineered strain that expressed an NADPH-dependent alcohol dehydrogenase from *Entamoeba histolytica*, combined with overexpression of *S. cerevisiae* NADP-dependent glucose-6-P dehydrogenase (Zwf1, EC 1.1.1.49: glucose-6-phosphate + NADP^+^ → 6-phospho-glucono-1,5-lactone + NADPH) and acetyl-CoA synthetase (Acs2) showed an almost 3-fold higher acetate consumption [95].

A different strategy to increase the potential for acetate reduction by A-ALD expressing strains is to enable anaerobic co-conversion of glycerol, which is left in the final phases of fermentation or obtained from post-distillation stills [96], to ethanol. In the patent literature, an NADH-specific glycerol dehydrogenase from *E. coli* (*gldA*, EC 1.1.1.6: glycerol + NAD^+^ → dihydroxyacetone + NADH + H^+^) was expressed together with an additional copy of *DAK1*, encoding dihydroxyacetone kinase (EC 2.7.1.29: dihydroxyacetone + ATP → dihydroxyacetone phosphate + ADP) [97,98]. Combined with enzymes from the lower half of glycolysis, pyruvate decarboxylase and alcohol dehydrogenase, GldA and Dak1 enable conversion of glycerol to ethanol with the formation of 1 mol of NADH (Fig. 3H). When, besides sugars, glycerol and acetate are present as an additional substrates in A-ALD expressing cultures, glycerol conversion to ethanol acts as source of NADH enabling more acetate reduction. Indeed, high apparent ethanol yields of 0.48–0.50 g ethanol per gram of glucose were reported for *S. cerevisiae* strains in which *gldA* and *DAK1* overexpression was combined with expression of *E. coli mhpF* or *EutE* [97,98].

### 4.4 Integration of acetyl-CoA reduction by A-ALD in yeast sugar metabolism

Organic acid concentrations in ‘first generation’ feedstocks for yeast-based ethanol production are generally around 1.3 g/L, [37,99], which limits the potential impact of the replacement of glycerol production by reduction of exogenous acetate via an engineered A-ALD pathway. In such settings, NADH re-oxidation by A-ALD could still replace glycerol production if acetyl-CoA is formed from glucose by pathways that yield fewer than 2 mol of NADH per mole of acetyl-CoA. The patent literature describes two strategies to achieve this goal, of which the first is based on heterologous expression of a bacterial pyruvate formate-lyase (PFL; EC 2.3.1.54: pyruvate → acetyl-CoA + formate) in A-ALD-expressing *S. cerevisiae* [100,101] (Fig. 3E). PFL, which is an oxygen-sensitive enzyme, was shown to be able to functionally replace the native pathway for acetyl-CoA synthesis in anaerobic *S. cerevisiae* cultures [102,103]. Synthesis of acetyl-CoA via glycolysis and PFL yields only one NADH per acetyl-CoA and thus enables a net reduction of one NADH when combined with ethanol production via A-ALD and yeast alcohol dehydrogenase. To prevent NADH formation by the yeast formate dehydrogenases Fdh1 and Fdh2 (EC 1.17.1.9: formate + NAD^+^ → CO_2_ + NADH; [104]), it was proposed to delete *FDH1* and *FDH2* from PFL/A-ALD expressing strains [100,101].

A second strategy for coupling A-ALD to sugar metabolism proposed in the patent literature [105] is based generation of acetyl-CoA through phosphoketolase (EC 4.1.2.9) and phosphotransacetylase (EC 2.3.1.8) (Fig. 3F). In this strategy, xylulose-5-phosphate is first formed from glucose in a redox-cofactor neutral manner via the enzymes of the non-oxidative pentose-phosphate pathway. This sugar phosphate is then converted into glyceraldehyde-3-phosphate and acetyl-phosphate by a heterologously expressed phosphoketolase (PK, EC 4.1.2.9: xylulose-5-phosphate + P_i_ → acetyl-phosphate + glyceraldehyde-3-phosphate + H_2_O). Subsequently, a heterologously expressed phosphotransacetylase (PTA, EC 2.3.1.8: acetyl-phosphate + CoA → acetyl-CoA + P_i_) converts acetyl phosphate to acetyl-CoA. This pathway has been successfully used for the ATP-efficient generation of acetyl-CoA as a precursor for aerobic product formation by engineered *S. cerevisiae* strains [106,107]. While the exact impact on ethanol yields will depend on strain and process characteristics, both pathways have the theoretical potential to completely replace the role of glycerol formation in NADH re-oxidation.

### 4.5 Expression of Calvin-cycle enzymes

Phosphoribulokinase (PRK, EC 2.7.1.19: ribulose-5-phosphate + ATP → ribulose-1,5-biphosphate + ADP) and ribulose-1,5-bisphosphate carboxylase/oxygenase (Rubisco, EC 4.1.1.39: ribulose-1,5-biphosphate + CO_2_ + H_2_O → 2 glyceraldehyde-3-phosphate + 2H^+^) are the two key enzymes of the Calvin cycle for autotrophic CO_2_ fixation. By capturing CO_2_, these enzymes together have the potential to generate a redox-cofactor-neutral bypass of the oxidative glyceraldehyde-3-phosphate dehydrogenase reaction in glycolysis when ribulose-5-phosphate, the substrate of phosphoribulokinase, is generated from glucose via the reactions of the non-oxidative pentose-phosphate pathway (Fig. 3D). In theory, this bypass should enable the use of ethanol formation as a redox sink for NADH generated in biosynthetic reactions. This hypothesis was tested by Guadalupe-Medina et al. (2013) [108], who demonstrated the presence of a functionally active Rubisco in cell extracts of an engineered *S. cerevisiae* strain that co-expressed the *Thiobacillus denitrificans* type-II Rubisco CbbM with the *E. coli* chaperonins GroEL and GroES. Co-expression of CbbM, GroEL, GroES with spinach phosphoribulokinase was shown to result in a 90% lower glycerol yield and a 10% higher ethanol yield in anaerobic, sugar-limited chemostat cultures grown at a dilution rate of 0.05 h^−1^ and sparged with CO_2_-enriched nitrogen [108]. In line with the low affinity of CbbM for CO_2_ [109], a less pronounced effect on glycerol and ethanol yields was observed when cultures were sparged with pure nitrogen gas.

Papapetridis et al. (2018) observed that an *S. cerevisiae* strain that combined constitutive expression of PRK, Rubisco, GroEL and GroES showed only a modest reduction of glycerol in fast-growing anaerobic batch cultures on glucose than the slow-growing chemostat cultures studied by Guadalupe-Medina et al. (2013). To improve competition of the Rubisco pathway for NADH with the native glycerol pathway, *GPD2* was deleted and the four key enzymes of the non-oxidative pentose phosphate pathway were overexpressed. In addition, PRK was expressed from a weaker, anaerobically inducible promoter to avoid reported toxic effects of PRK overexpression in microorganisms [110,111] during aerobic pre-cultivation. The resulting strain retained a wild-type growth rate in anaerobic, glucose-grown batch cultures, while showing an 86% lower glycerol yield and 15% higher ethanol yield on glucose than a congenic reference strain [77].

The strategies discussed above were first designed to reduce or eliminate the need for glycerol formation in alcoholic fermentation of disaccharides or hexoses. However, they can similarly be employed in conversion of other sugars into ethanol. Xylose-utilizing *S. cerevisiae* have been engineered either based on the functional expression of the fungal xylose reductase (XR, EC 1.1.1.307: xylose + NAD(P)H → xylitol + NAD(P)^+^) and xylitol dehydrogenase (XDH, EC 1.1.1.9: xylitol + NAD^+^ → xylulose + NADH), or the expression of a bacterial xylose isomerase (EC 5.3.1.5: xylose → xylulose). A key challenge in the strategy based on XR and XDH is that XR typically prefers NADPH as cofactor, while XDH exclusively uses NAD^+^ [112]. As a consequence of this cofactor imbalance, xylitol is formed as a byproduct. Changing the cofactor preference of ammonium assimilation as demonstrated by Nissen et al. (2000) [43] facilitated re-oxidation of NADH generated in the XDH reaction and improved ethanol yield in an XR/XDH-based *S. cerevisiae* strain [113]. Combined functional expression, of PRK and Rubisco [114,115]; phosphoketolase and phosphotransacetylase [116]; or GAPN [64] were similarly applied to improve redox co-factor balancing in XR/XDH-based strains and, thereby, ethanol yields on xylose.

## 5 Model-based comparison of maximum theoretical impact of individual engineering strategies

Experimentally determined ethanol yields achieved with the pathway engineering strategies discussed in paragraphs 3 and 4 (Table 1) can be influenced by experimental conditions as well as by the *S. cerevisiae* genetic background into which genetic modifications were introduced, for example due to different biomass compositions. To eliminate these factors, different pathway strategies were implemented in a stoichiometric model of the core metabolic network of *S. cerevisiae* [117] and used to calculate growth stoichiometries of anaerobic, sugar-grown cultures (Table 2). Although the resulting estimates cannot be used to predict performance of strategies in specific strain backgrounds or processes, they do enable comparison of the maximum impact of the different strategies and identification of trade-offs.

**Table 1:** Reported impacts on glycerol production, maximum specific growth rate and ethanol production in anaerobic batch cultures of *S. cerevisiae* strains subjected to different pathway engineering strategies aimed at reducing glycerol production and improving ethanol yield. Depending on the studies, changes in product yields were either expressed per amount of substrate or per amount of biomass. Subscript x denotes dry biomass, ↑ indicates overexpression of native *S. cerevisiae* genes.

Strategy	Genotype	Glycerol yield	Growth rate	Ethanol yield	Reference
Altered cofactor specificity of ammonium assimilation	*gdh1Δ GLN1↑GLT1↑*	−38% (g/g glucose)	−10%	+10% (g/g glucose)	[18]
	*gdh1Δ GDH2↑*	−30% (g/g glucose)	−5%	+3% (g/g glucose)	[18]
NADH-dependent reduction of acetate to ethanol	*gpd1Δ gpd2Δ* Ec-*mphF*	−100% (g/g_x_)	−56%	+13% (g/g glucose)	[90]
(Ec = *E. coli*)	*gpd1Δ gpd2Δ* Ec-*eutE*	−100% (g/g_x_)	−7%	+9% (g/g glucose)	[94]
NADH-dependent reduction of acetate to ethanol with increased NADH generation via pentose-phosphate pathway	*gnd2Δ gnd1Δ gndAΔ ald6Δ gpd1Δ gpd2Δ* Ec-*eutE*	−100% (g/g_x_)	−29%	+11% (g/g glucose)	[94]
NADH re-oxidation via expression of Calvin-cycle enzymes, optimized for anaerobic growth rate (So = *Spinacia oleracea*, Td = *Thiobacillus denitrificans*)	*gpd2Δ RPE1↑TKL1↑ TAL1↑ NQM1↑ RKI1↑ TKL2↑* So*-prk* Td*-cbbm* (9 copies) Ec-*groES,* Ec-*groEL*	−86% (g/g_x_)	0%	+15% (g/g glucose)	[77]
Reduced NADH and ATP formation in glycolysis by expression of *gapN*	Sm-*gapN*	−40% (g/g glucose)	0%	+2% (g/g glucose)	[64]
(Sm = *Streptococcus mutans*)	*gpd1Δ* Sm-*gapN TPS1↑ TPS2↑*	−73% (g/g glucose)	0%	+8% (g/g glucose)	[62]

**Table 2:** Maximum impact of different pathway engineering strategies for improving ethanol yields, estimated with a stoichiometric model of the core metabolic network of *S. cerevisiae* [117]. Assumptions on biomass composition, maintenance-energy requirements, as well as modifications to the model that were implemented to simulate each of the metabolic engineering strategies, are described in Supplementary Materials. For the strategies focused on NADH re-oxidation, glycerol production was set at zero and oxidation of surplus NADH from biosynthetic reactions was entirely routed through the engineered pathways.

Specific growth rate (h^−1^)	Y_ethanol/hexose_ (mol/mol)					
	Reference	Altered ATP coupling of sugar dissimilation		Alternative pathways for re-oxidation of NADH		
	Wild type	H^+^ symport/intracellular hydrolysis of sucrose (yields 1.5 ATP/hexose)	H^+^ symport of glucose (yields 1 ATP/glucose)	PFL/A-ALD	PK/PTA/A-ALD	PRK/Rubisco
0.3	1.51	1.63 (8.1%)	1.76 (16.2%)	1.64 (8.7%)	1.66 (9.7%)	1.69 (11.9%)
0.1	1.54	1.66 (7.5%)	1.77 (14.9%)	1.67 (8.4%)	1.69 (9.5%)	1.71 (11.3%)
0.03	1.62	1.72 (5.8%)	1.81 (11.6%)	1.74 (7.4%)	1.76 (8.5%)	1.78 (9.5%)
0.01	1.75	1.81 (3.6%)	1.87 (7.2%)	1.84 (5.2%)	1.86 (6.1%)	1.86 (6.4%)
0.001	1.95	1.97 (0.6%)	1.98 (1.2%)	1.97 (1.0%)	1.98 (1.2%)	1.98 (1.2%)

To evaluate pathway engineering strategies aimed at reducing the ATP yield from sugar fermentation, two scenarios were simulated. In the first, glucose import required a net input of 0.5 ATP, which corresponds to the ATP yield per hexose unit in strains that combine sucrose-proton symport with intracellular sucrose hydrolysis [70]. The second scenario, in which glucose import required 1 ATP, corresponds to a situation in which hexose transport occurs via symport with a proton or, alternatively, glucose is fermented via an alternative glycolytic pathway with a net ATP yield of 1 mol/mol glucose (e.g. the Entner-Doudoroff pathway) in combination with a glucose facilitator. At a specific growth rate of 0.30 h^−1^, simulation of these scenarios gave predicted increases of ethanol yield on hexose equivalents of 8.1% and 16.2%, respectively (Table 2). Due to a larger impact of a constant maintenance-energy requirement at low growth rate [22,118], predicted benefits of these engineering strategies declined as the specific growth rate approached zero (Table 2). An important consequence of these two strategies was that, at each specific growth rate, specific rates of sugar conversion were 33% and 100% higher, respectively, than in the reference situation (Supplementary Table 1). Especially at high specific growth rates, which are important for supporting high volumetric productivities in industrial batch processes, achieving such high conversion rates may be challenging due to the requirement for a large resource allocation to glycolytic proteins [119,120] or for membrane space to accommodate the required number of sugar transporters [121]. In addition, concomitant reductions of the biomass yield on sugar by 25% and 50%, respectively (Supplementary Table 2) may cause economic trade-offs when surplus yeast biomass is sold as a co-product for application in animal feed products [12].

To assess the maximum theoretical impact on ethanol yield of the strategies focused on redox-cofactor balancing, glycerol production was set to zero, so that re-oxidation of NADH generated in biosynthesis occurred exclusively via the engineered pathways. At a specific growth rate of 0.3 h^−1^, the PFL/A-ALD, PK/PTA/A-ALD and PRK/Rubisco strategies yielded predicted improvements of the ethanol yield on glucose of 8.7%, 9.7% and 11.9%, respectively. The predicted differences between the impacts of the three strategies can be predominantly attributed to the different net ATP and ethanol yields for NADH re-oxidation via these pathways. Due to different ATP and carbon efficiencies of these heterologous pathways, implementation of these redox engineering strategies in the stoichiometric model also led to higher predicted biomass yields on glucose and correspondingly lower specific rates of glucose consumption (Supplementary Tables 1 and 2). Thus, in contrast to strategies aimed at reducing the ATP stoichiometry of sugar fermentation, their industrial implementation should not be affected by a potentially limited capacity of sugar fermentation and/or transport or by a trade-off with revenues from surplus yeast biomass. As observed for the strategies aimed at engineering ATP coupling of sugar dissimilation, the impact of the redox-engineering strategies on ethanol yield declined with decreasing specific growth rate and, at the lowest simulated growth rate (0.001 h^−1^), the predicted increase of ethanol yield on glucose was only approximately 1%.

For several of the strategies, experimental studies (Table 1) yielded larger improvements of the ethanol yield than the maximum theoretical improvements shown in Table 2. In addition to differences in biomass composition and ethanol yields of reference *S. cerevisiae* strains, these differences may reflect unintended impacts of genetic modifications on cellular energy requirements. For example, high-level expression of heterologous proteins has been associated with increased cellular energy requirements [122,123] which, in anaerobic cultures, can contribute to higher ethanol yields. In addition, alteration of the expression of membrane proteins may potentially lead to increased ATP dissipation, for exampling by futile cycling of glucose through overexpressed Mal11 and Hxt transporters.

## 6 Discussion and outlook

As outlined in this review, multiple pathway engineering strategies have been demonstrated to improve ethanol yields on sugars in anaerobic laboratory cultures of *S. cerevisiae* by altering the ratio of the formation of ethanol, biomass and glycerol. However, observations made under controlled conditions in laboratory-scale media are not necessarily representative for industrial processes. Even in anaerobic glucose-limited cultures of wild-type *S. cerevisiae*, ethanol yields on glucose approach the theoretical maximum of 2 mol ethanol/mol glucose at near-zero growth rates [22]. Consequently, predicted benefits of all investigated pathway engineering strategies strongly depend on specific growth rate (Table 2). In industrial batch processes, the impact of the described engineering strategies on ethanol yield is likely to be highest during the initial phase in which vigorous growth occurs. Conversely, during the final phases of a batch fermentation process, where growth has essentially ceased and high ethanol concentrations lead to an increased maintenance energy requirement, their impact may well be negligible.

In addition to the inherent dynamics of industrial processes, development of industrial strains should take into account trade-offs between ethanol yield and other performance indicators. In particular, an improved product yield should not go at the expense of productivity. With few exceptions, academic studies reported that *S. cerevisiae* strains which were successfully engineered for improved ethanol yield grew slower than their non-engineered parental strains (Table 1). The extensive synthetic biology toolbox for genetic modification of *S. cerevisiae*, including approaches such as multiplexed Cas9-mediated genome editing and *in vivo* assembly and chromosomal integration of synthetic DNA fragments [124,125], is therefore intensively used to explore options for maximizing ethanol yields by engineering its metabolic network. In addition, pathway engineering in this yeast benefits from the availability of genome-scale metabolic models (for reviews see Refs. [126,127]), which allow for fast predictions of the impact of genetic interventions on distribution of fluxes in metabolic networks. A dedicated study on PRK/Rubisco based strains [77] illustrates that restoring the specific growth rate of engineered strains to wild-type levels may require substantial additional engineering. Alternatively, adaptive laboratory evolution and/or reverse engineering of evolved strains [128,129] can be used for this purpose. Another important trade-off concerns cellular robustness. Until engineering strategies are available that fully restore osmotolerance in glycerol-negative strains, strategies aimed at reducing glycerol production should not completely eliminate glycerol production [76]. In addition to targeted engineering strategies, robustness may be increased by using natural and industrial *S. cerevisiae* strains with a high innate tolerance to industrially relevant stress factors in strain improvement programmes [130,131].

Temperature, pH, pCO_2_, ethanol concentration and their dynamics in large-scale industrial processes may affect the impact of engineering strategies, thus requiring process-specific strain optimization. The economic significance of small differences in ethanol yield, combined with the use of non-defined industrial media and dynamic industrial processes, raises non-trivial challenges in setting up high-throughput cultivation and analysis systems that faithfully predict strain performance in real-life applications. Although companies tend not to disclose the genetic make-up of industrial strains, the introduction of multiple ‘high-ethanol-yield’ *S. cerevisiae* strains into USA-based ethanol plants [[132], [133], [134]] indicates that at least some of the strategies discussed in this review already contribute to profitability and sustainability of industrial ethanol production. Introduction into Brazil, the second-largest ethanol-producing economy, may involve additional challenges related to the use of non-aseptically operated, extended production campaigns. This mode of operation not only poses high demands on the genetic stability of engineered strains, for example to prevent recovery of glycerol production by strains with down-regulated *GPD1* and/or *GPD2* expression, but also on their ability to compete with ‘wild’ strains entering the process [135].

Improving ethanol yield on fermentable sugars is by no means the only target of metabolic engineering studies related to yeast-based ethanol production. Other targets of intensive research include the reduction of processing costs by expression of polysaccharide hydrolases [136], extending substrate range to convert more fermentable substrates in crude industrial media [137,138], improving performance at high temperature to improve heat economy and cope with process temperature profiles [139], increasing yeast tolerance to process inhibitors and ethanol [139,140], improving osmotolerance of engineered strains with reduced glycerol formation [16,141] and simplification of nutritional requirements of industrial strains [[142], [143], [144], [145]]. In addition, integration of corn-fiber from 1.5G processes [21] and reducing the need for antibiotics [139,146] are actively explored. Combination of these and other relevant traits with strategies for improving product yield, in *S. cerevisiae* and potentially also in other yeast species [130,147,148] will, in the coming years, continue to generate interesting challenges for academic and industrial research.

## Author contributions

AA: Formal analysis, Investigation, Data Curation, Writing – Original Draft, Writing – Review & Editing SV: Investigation, Data Curation, Writing – Original Draft, Writing – Review & Editing, Visualization WG Formal analysis MJ: Writing – Review & Editing JP: Conceptualization, Writing – Original Draft, Writing – Review & Editing RM: Writing – Original Draft, Writing – Review & Editing, Project administration.

## Declaration of competing interest

The PhD project of AA is funded by DSM Bio-based Products & Services B.V. (Delft, The Netherlands). Royal DSM owns intellectual property rights of technology discussed in this paper.

## References

[1] Renewable Fuels Association (2020). https://ethanolrfa.org/markets-and-statistics/annual-ethanol-production.

[2] Mohsenzadeh A., Zamani A., Taherzadeh M.J. (2017). Bioethylene production from ethanol: a review and techno-economical evaluation. ChemBioEng Rev..

[3] Capaz R., Posada J., Seabra J., Osseweijer P. (2018). Proceedings of the 26th European biomass conference and exhibition; 2018 may 14-18; copenhagen, Denmark.

[4] Ruchala J., Kurylenko O.O., Dmytruk K.V., Sibirny A.A. (2020). Construction of advanced producers of first- and second-generation ethanol in *Saccharomyces cerevisiae* and selected species of non-conventional yeasts (*Scheffersomyces stipitis, Ogataea polymorpha*). J Ind Microbiol Biotechnol.

[5] Thomas K.C., Ingledew W.M. (1992). Production of 21% (v/v) ethanol by fermentation of very high gravity (VHG) wheat mashes. J Ind Microbiol.

[6] Devantier R., Pedersen S., Olsson L. (2005). Characterization of very high gravity ethanol fermentation of corn mash. Effect of glucoamylase dosage, pre-saccharification and yeast strain. Appl Microbiol Biotechnol.

[7] Pfromm P.H., Amanor-Boadu V., Nelson R., Vadlani P., Madl R. (2010). Bio-butanol vs. bio-ethanol: a technical and economic assessment for corn and switchgrass fermented by yeast or Clostridium acetobutylicum. Biomass Bioenergy.

[8] Lian J., Mishra S., Zhao H. (2018). Recent advances in metabolic engineering of *Saccharomyces cerevisiae*: new tools and their applications. Metab Eng.

[9] de Smidt O., du Preez J.C., Albertyn J. (2008). The alcohol dehydrogenases of *Saccharomyces cerevisiae*: a comprehensive review. FEMS Yeast Res.

[10] Roels J.A. (1980). A simple model for the energetics of growth on substrates with different degrees of reduction. Biotechnol Bioeng.

[11] Russell J.B., Cook G.M. (1995). Energetics of bacterial growth: balance of anabolic and catabolic reactions. Microbiol Rev.

[12] Conroy B.B., Bittner C.J., Donald J.C.M., Luebbe M.K., Erickson G.E. (2016).

[13] Verduyn C., Postma E., Scheffers W.A., van Dijken J.P. (1990). Physiology of *Saccharomyces cerevisiae* in anaerobic glucose-limited chemostat cultures. J Gen Microbiol.

[14] Bakker B.M., Overkamp K.M., van Maris A.J.A., Kötter P., Luttik M.A.H., van Dijken J.P., Pronk J.T. (2001). Stoichiometry and compartmentation of NADH metabolism in Saccharomyces cerevisiae. FEMS (Fed Eur Microbiol Soc) Microbiol Rev.

[15] Eriksson P., André L., Ansell R., Blomberg A., Adler L. (1995). Cloning and characterization of *GPD2*, a second gene encoding *sn*-glycerol 3-phosphate dehydrogenase (NAD^+^) in *Saccharomyces cerevisiae*, and its comparison with *GPD1*. Mol Microbiol.

[16] Albertyn J., Hohmann S., Thevelein J.M., Prior B.A. (1994). *GPD1*, which encodes glycerol-3-phosphate dehydrogenase, is essential for growth under osmotic stress in *Saccharomyces cerevisiae*, and its expression is regulated by the high-osmolarity glycerol response pathway. Mol Cell Biol.

[17] Norbeck J., Påhlman A.-K., Akhtar N., Blomberg A., Adler L. (1996). Purification and characterization of two isoenzymes of DL-glycerol-3-phosphatase from Saccharomyces cerevisiae. J Biol Chem.

[18] Nissen T.L., Hamann C.W., Kielland-Brandt M.C., Nielsen J., Villadsen J. (2000). Anaerobic and aerobic batch cultivations of *Saccharomyces cerevisiae* mutants impaired in glycerol synthesis. Yeast.

[19] den Haan R., Kroukamp H., Mert M., Bloom M., Görgens J.F., van Zyl W.H. (2013). Engineering *Saccharomyces cerevisiae* for next generation ethanol production. Chem Technol Biotechnol.

[20] Laluce C., Schenberg A.C.G., Gallardo J.C.M., Coradello L.F.C., Pombeiro-Sponchiado S.R. (2012). Advances and developments in strategies to improve strains of *Saccharomyces cerevisiae* and processes to obtain the lignocellulosic Ethanol−A review. Appl Biochem Biotechnol.

[21] Jansen M.L.A., Bracher J.M., Papapetridis I., Verhoeven M.D., de Bruin H., de Waal P.P., van Maris A.J.A., Klaassen P., Pronk J.T. (2017). *Saccharomyces cerevisiae* strains for second-generation ethanol production: from academic exploration to industrial implementation. FEMS Yeast Res.

[22] Boender L.G.M., de Hulster E., van Maris A.J.A., Daran-Lapujade P., Pronk J.T. (2009). Quantitative physiology of *Saccharomyces cerevisiae* at near-zero specific growth rates. Appl Environ Microbiol.

[23] Vos T., de la Torre Cortés P., van Gulik W.M., Pronk J.T., Daran-Lapujade P. (2015). Growth-rate dependency of de novo resveratrol production in chemostat cultures of an engineered *Saccharomyces cerevisiae* strain. Microb Cell Factories.

[24] Tännler S., Decasper S., Sauer U. (2008). Maintenance metabolism and carbon fluxes in *Bacillus* species. Microb Cell Factories.

[25] Abbott D.A., Suir E., van Maris A.J.A., Pronk J.T. (2008). Physiological and transcriptional responses to high concentrations of lactic acid in anaerobic chemostat cultures of Saccharomyces cerevisiae. Appl Environ Microbiol.

[26] Viegas C.A., Sá-Correia I. (1991). Activation of plasma membrane ATPase of *Saccharomyces cerevisiae* by octanoic acid. J Gen Microbiol.

[27] Taherzadeh M.J., Niklasson C., Lidén G. (1997). Acetic acid - friend or foe in anaerobic batch conversion of glucose to ethanol by *Saccharomyces cerevisiae*?. Chem Eng Sci.

[28] Verduyn C., Postma E., Scheffers W.A., van Dijken J.P. (1992). Effect of benzoic acid on metabolic fluxes in yeasts: a continuous-culture study on the regulation of respiration and alcoholic fermentation. Yeast.

[29] Verduyn C., Postma E., Scheffers W.A., van Dijken J.P. (1990). Energetics of *Saccharomyces cerevisiae* in anaerobic glucose-limited chemostat cultures. J Gen Microbiol.

[30] Narendranath N.V., Thomas K.C., Ingledew W.M. (2001). Acetic acid and lactic acid inhibition of growth of *Saccharomyces cerevisiae* by different mechanisms. J Am Soc Brew Chem.

[31] Weusthuis R.A., Adams H., Scheffers W.A., van Dijken J.P. (1993). Energetics and kinetics of maltose transport in *Saccharomyces cerevisiae*: a continuous culture study. Appl Environ Microbiol.

[32] Serrano R. (1977). Energy requirements for maltose transport in yeast. Eur J Biochem.

[33] Cardoso H., Leão C. (1992). Mechanisms underlying the low and high enthalpy death induced by short-chain monocarboxylic acids and ethanol in Saccharomyces cerevisiae. Appl Microbiol Biotechnol.

[34] Santos J., Sousa M.J., Cardoso H., Inácio J., Silva S., Spencer-Martins I., Leão C. (2008). Ethanol tolerance of sugar transport, and the rectification of stuck wine fermentations. Microbiology.

[35] Dunlop A.P. (1948). Furfural formation and behavior. Ind Eng Chem.

[36] Ulbricht R.J., Northup S.J., Thomas J.A. (1984). A review of 5-hydroxymethylfurfural (HMF) in parental solutions. Fund Appl Toxicol.

[37] Russell I., Jacques K.A., Lyons T.P., Kelsall D.R. (2003). The alcohol textbook.

[38] Monteiro G.A., Supply P., Goffeau A., Sá-Correia I. (1994). The *in vivo* activation of *Saccharomvces cerevisiae* plasma membrane H^+^-ATPase by ethanol depends on the expression of the *PMA1* gene, but not of the *PMA2* gene. Yeast.

[39] Rosa M.F., Sá-Correia I. (1991). In vivo activation by ethanol of plasma membrane ATPase of Saccharomyces cerevisiae. Appl Environ Microbiol.

[40] Lagunas R. (1993). Sugar transport in Saccharomyces cerevisiae. FEMS (Fed Eur Microbiol Soc) Microbiol Rev.

[41] Boles E., Hollenberg C.P. (1997). The molecular genetics of hexose transport in yeasts. FEMS (Fed Eur Microbiol Soc) Microbiol Rev.

[42] Van Leeuwen C.C.M., Weusthuis R.A., Postma E., Van den Broek P.J.A., Van Dijken J.P. (1992). Maltose/proton co-transport in *Saccharomyces cerevisiae*. Comparative study with cells and plasma membrane vesicles. Biochem J.

[43] Nissen T.L., Kielland-Brandt M.C., Nielsen J., Villadsen J. (2000). Optimization of ethanol production in Saccharomyces cerevisiae by metabolic engineering of the ammonium assimilation. Metab Eng.

[44] Nissen T.L., Schulze U., Nielsen J., Villadsen J. (1997). Flux distributions in anaerobic, glucose-limited continuous cultures of Saccharomyces cerevisiae. Microbiology.

[45] Albers E., Larsson C., Lidén G., Niklasson C., Gustafsson L. (1996). Influence of the nitrogen source on *Saccharomyces cerevisiae* anaerobic growth and product formation. Appl Environ Microbiol.

[46] Radler F., Schütz H. (1982). Glycerol production of various strains of Saccharomyces. Am J Enol Vitic.

[47] Jørgensen H. (2009). Effect of nutrients on fermentation of pretreated wheat straw at very high dry matter content by Saccharomyces cerevisiae. Appl Biochem Biotechnol.

[48] Zahoor A., Messerschmidt K., Boecker S., Klamt S. (2020). ATPase-based implementation of enforced ATP wasting in *Saccharomyces cerevisiae* for improved ethanol production. Biotechnol Biofuels.

[49] Jensen P.R., Snoep J.L., Westerhoff H.V., inventor; Peter Ruhdal Jensen, assignee (2007 July 31).

[50] Semkiv M.V., Dmytruk K.V., Abbas C.A., Sibirny A.A. (2014). Increased ethanol accumulation from glucose via reduction of ATP level in a recombinant strain of *Saccharomyces cerevisiae* overexpressing alkaline phosphatase. BMC Biotechnol.

[51] Rogers D.T., Szostak J.W., inventor; Genetics Institute, Inc., assignee (1993 Dec 7).

[52] Navas M.A., Cerdán S., Gancedo C. (1993). Futile cycles in *Saccharomyces cerevisiae* strains expressing the gluconeogenic enzymes during growth on glucose. Proc Natl Acad Sci Unit States Am.

[53] Navas M.A., Gancedo C. (1996). The regulatory characteristics of yeast fructose-1,6-bisphosphatase confer only a small selective advantage. J Bacteriol.

[54] Semkiv M.V., Dmytruk K.V., Abbas C.A., Sibirny A.A. (2016). Activation of futile cycles as an approach to increase ethanol yield during glucose fermentation in Saccharomyces cerevisiae. Bioengineered.

[55] Rogers P.L., Lee K.J., Tribe D.E. (1979). Kinetics of alcohol production by *Zymomonas mobilis* at high sugar concentrations. Biotechnol Lett.

[56] Lee K.J., Skotnicki M.L., Tribe D.E., Rogers P.L. (1980). Kinetic studies on a highly productive strain of Zymomonas mobilis. Biotechnol Lett.

[57] Lancashire W.E., Dickinson J.R., Malloch R.A. (1994 Jul 28).

[58] Benisch F., Boles E. (2014). The bacterial Entner–Doudoroff pathway does not replace glycolysis in *Saccharomyces cerevisiae* due to the lack of activity of iron–sulfur cluster enzyme 6-phosphogluconate dehydratase. J Biotechnol.

[59] Morita K., Nomura Y., Ishii J., Matsuda F., Kondo A., Shimizu H. (2017). Heterologous expression of bacterial phosphoenol pyruvate carboxylase and Entner-Doudoroff pathway in *Saccharomyces cerevisiae* for improvement of isobutanol production. J Biosci Bioeng.

[60] Gardner P.R., Fridovich I. (1991). Superoxide sensitivity of *Escherichia coli* 6-phosphogluconate dehydratase. J Biol Chem.

[61] Biz A., Mahadevan R. (2021). Overcoming challenges in expressing iron–sulfur enzymes in yeast. Trends Biotechnol.

[62] Guo Z-p, Zhang L., Ding Z-y, Shi G-y (2011). Minimization of glycerol synthesis in industrial ethanol yeast without influencing its fermentation performance. Metab Eng.

[63] Zhang L., Tang Y., Guo Z-p, Ding Z-y, Shi G-y (2011). Improving the ethanol yield by reducing glycerol formation using cofactor regulation in Saccharomyces cerevisiae. Biotechnol Lett.

[64] Bro C., Regenberg B., Förster J., Nielsen J. (2006). In silico aided metabolic engineering of *Saccharomyces cerevisiae* for improved bioethanol production. Metab Eng.

[65] Gascón S., Lampen J.O. (1968). Purification of the internal invertase of yeast. J Biol Chem.

[66] Carlson M., Botstein D. (1982). Two differentially regulated mRNAs with different 5' ends encode secreted and intracellular forms of yeast invertase. Cell.

[67] Kruckeberg A.L. (1996). The hexose transporter family of Saccharomyces cerevisiae. Arch Microbiol.

[68] Stambuk B.U., Batista A.S., de Araujo P.S. (2000). Kinetics of active sucrose transport in Saccharomyces cerevisiae. J Biosci Bioeng.

[69] Santos E., Rodriguez L., Elorza M.V., Sentandreu R. (1982). Uptake of sucrose by Saccharomyces cerevisiae. Arch Biochem Biophys.

[70] Basso T.O., de Kok S., Dario M., do Espirito-Santo J.C.A., Müller G., Schlölg P.S., Silva C.P., Tonso A., Daran J.-M.G., Gombert A.K., Van Maris A.J.A., Pronk J.T., Stambuk B.U. (2011). Engineering topology and kinetics of sucrose metabolism in *Saccharomyces cerevisiae* for improved ethanol yield. Metab Eng.

[71] de Kok S., Kozak B.U., Pronk J.T., van Maris A.J.A. (2012). Energy coupling in *Saccharomyces cerevisiae*: selected opportunities for metabolic engineering. FEMS Yeast Res.

[72] Tiukova I.A., Møller-Hansen I., Belew Z.M., Darbani B., Boles E., Nour-Eldin H.H., Linder T., Nielsen J., Borodina I. (2019). Identification and characterisation of two high-affinity glucose transporters from the spoilage yeast Brettanomyces bruxellensis. FEMS (Fed Eur Microbiol Soc) Microbiol Lett.

[73] Athenstaedt K., Weys S., Paltauf F., Daum G. (1999). Redundant systems of phosphatidic acid biosynthesis via acylation of glycerol-3-phosphate or dihydroxyacetone phosphate in the yeast Saccharomyces cerevisiae. J Bacteriol.

[74] Ansell R., Granath K., Hohmann S., Thevelein J.M., Adler L. (1997). The two isoenzymes for yeast NAD^+^‐dependent glycerol 3‐phosphate dehydrogenase encoded by *GPD1* and *GPD2* have distinct roles in osmoadaptation and redox regulation. EMBO J.

[75] Björkqvist S., Ansell R., Adler L., Liden G. (1997). Physiological response to anaerobicity of glycerol-3-phosphate dehydrogenase mutants of Saccharomyces cerevisiae. Appl Environ Microbiol.

[76] Blomberg A., Adler L. (1992). Physiology of osmotolerance in fungi. Adv Microb Physiol.

[77] Papapetridis I., Goudriaan M., Vázques Vitali M., de Keijzer N.A., Van den Broek M., van Maris A.J.A., Pronk J.T. (2018). Optimizing anaerobic growth rate and fermentation kinetics in *Saccharomyces cerevisiae* strains expressing Calvin-cycle enzymes for improved ethanol yield. Biotechnol Biofuels.

[78] van Dijken J.P., Scheffers W.A. (1986). Redox balances in the metabolism of sugars by yeasts. FEMS (Fed Eur Microbiol Soc) Microbiol Rev.

[79] Henriques D., Minebois R., Mendoza S.N., Macías L.G., Pérez-Torrado R., Barrio E., Teusink B., Querol A., Balsa-Canto E. (2021). A multiphase multiobjective dynamic genome-scale model shows different redox balancing among yeast species of the *Saccharomyces* genus in fermentation. mSystems.

[80] Hubmann G., Guillouet S., Nevoigt E. (2011). Gpd1 and Gpd2 fine-tuning for sustainable reduction of glycerol formation in Saccharomyces cerevisiae. Appl Environ Microbiol.

[81] Bruinenberg P.M., van Dijken J.P., Scheffers W.A. (1983). A theoretical analysis of NADPH production and consumption in yeasts. J Gen Microbiol.

[82] Bruinenberg P.M., Jonker R., van Dijken J.P., Scheffers W.A. (1985). Utilization of formate as an additional energy source by glucose-limited chemostat cultures of *Candida utilis* CBS 621 and *Saccharomyces cerevisiae* CBS 8066. Arch Microbiol.

[83] Anderlund M., Nissen T.L., Nielsen J., Villadsen J., Rydström J., Hahn-Hägerdal B., Kielland-Brandt M.C. (1999). Expression of the *Escherichia coli pntA* and *pntB* genes, encoding nicotinamide nucleotide transhydrogenase, in *Saccharomyces cerevisiae* and its effect on product formation during anaerobic glucose fermentation. Appl Environ Microbiol.

[84] Nissen T.L., Anderlund M., Nielsen J., Villadsen J., Kielland‐Brandt M.C. (2001). Expression of a cytoplasmic transhydrogenase in *Saccharomyces cerevisiae* results in formation of 2‐oxoglutarate due to depletion of the NADPH pool. Yeast.

[85] Nogae I., Johnston M. (1990). Isolation and characterisation of the *ZWF1* gene of *Saccharomyces cerevisiae,* encoding glucose-6-phosphate dehydrogenase. Gene.

[86] Vanrolleghem P.A., de Jong-Gubbels P., van Gulik W.M., Pronk J.T., van Dijken J.P., Heijnen J.J. (1996). Validation of a metabolic network for *Saccharomyces cerevisiae* using mixed substrate studies. Biotechnol Prog.

[87] Navarrete C., Nielsen J., Siewers V. (2014). Enhanced ethanol production and reduced glycerol formation in *fps1Δ* mutants of *Saccharomyces cerevisiae* engineered for improved redox balancing. Amb Express.

[88] Remize F., Barnavon L., Dequin S. (2001). Glycerol export and glycerol-3-phosphate dehydrogenase, but not glycerol phosphatase, are rate limiting for glycerol production in Saccharomyces cerevisiae. Metab Eng.

[89] Ciriacy M. (1975). Genetics of alcohol dehydrogenase in *Saccharomyces cerevisiae.* I. Isolation and genetic analysis of *adh* mutants. Mutat Res.

[90] Guadalupe Medina V., Almering M.J., van Maris A.J., Pronk J.T. (2010). Elimination of glycerol production in anaerobic cultures of a Saccharomyces cerevisiae strain engineered to use acetic acid as an electron acceptor. Appl Environ Microbiol.

[91] van den Berg M.A., de Jong-Gubbels P., Kortland C.J., van Dijken J.P., Pronk J.T., Steensma H.Y. (1996). The two acetyl-coenzyme A synthetases of *Saccharomyces cerevisiae* differ with respect to kinetic properties and transcriptional regulation. J Biol Chem.

[92] Papapetridis I., van Dijk M., van Maris A.J., Pronk J.T. (2017). Metabolic engineering strategies for optimizing acetate reduction, ethanol yield and osmotolerance in Saccharomyces cerevisiae. Biotechnol Biofuels.

[93] Almeida J.R.M., Modig T., Petersson A., Hähn-Hägerdal B., Lidén G., Gorwa-Grauslund M. (2007). Increased tolerance and conversion of inhibitors in lignocellulosic hydrolysates by Saccharomyces cerevisiae. J Chem Technol Biotechnol.

[94] Papapetridis I., van Dijk M., Dobbe A.P., Metz B., Pronk J.T., van Maris A.J. (2016). Improving ethanol yield in acetate-reducing *Saccharomyces cerevisiae* by cofactor engineering of 6-phosphogluconate dehydrogenase and deletion of *ALD6*. Microb Cell Factories.

[95] Henningsen B.M., Hon S., Covalla S.F., Sonu C., Argyros D.A., Barrett T.F., Wiswall E., Froehlich A.C., Zelle R.M. (2015). Increasing anaerobic acetate consumption and ethanol yields in *Saccharomyces cerevisiae* with NADPH-specific alcohol dehydrogenase. Appl Environ Microbiol.

[96] Kim Y., Mosier N.S., Hendrickson R., Ezeji T., Blaschek H., Dien B., Cotta M., Dale B., Ladisch M.R. (2008). Composition of corn dry-grind ethanol by-products: DDGS, wet cake, and thin stillage. Bioresour Technol.

[97] de Bont J.A.M., Teunissen A.W.R.H., Klaasen P., Hartman W.W.A., van Beusekom S. (2018 Jun 5).

[98] Klaassen P., Hartman W.W.A., inventor; DSM IP ASSETS B.V., assignee (2019 Oct 22).

[99] Rasmussen M.L., Koziel J.A., Jane J.-L., Pometto A.L. (2015). Reducing bacterial contamination in fuel ethanol fermentations by ozone treatment of uncooked corn mash. J Agric Food Chem.

[100] de Bont J.A.M., Teunissen A.W.R.H., inventor; Yeast Company B.V., assignee (2012 May 24).

[101] Argyros A., Sillers W.R., Barrett T., Caiazza N., Shaw A.J.I. (2015 Feb 17).

[102] Kozak B.U., van Rossum H.M., Benjamin K.R., Wu L., Daran J.-M.G., Pronk J.T., van Maris A.J. (2014). Replacement of the *Saccharomyces cerevisiae* acetyl-CoA synthetases by alternative pathways for cytosolic acetyl-CoA synthesis. Metab Eng.

[103] van Rossum H.M., Kozak B.U., Pronk J.T., van Maris A.J.A. (2016). Engineering cytosolic acetyl-coenzyme A supply in *Saccharomyces cerevisiae*: pathway stoichiometry, free-energy conservation and redox-cofactor balancing. Metab Eng.

[104] Overkamp K.M., Kötter P., van der Hoek R., Schoondermark-Stolk S., Luttik M.A.H., van Dijken J.P. (2002). Functional analysis of structural genes for NAD^+^-dependent formate dehydrogenase in Saccharomyces cerevisiae. Yeast.

[105] Andrei M., Munos J.W. (2017 Mar 30).

[106] Bergman A., Siewers V., Nielsen J., Chen Y. (2016). Functional expression and evaluation of heterologous phosphoketolases in Saccharomyces cerevisiae. Amb Express.

[107] Meadows A.L., Hawkins K.M., Tsegaye Y., Antipov E., Kim Y., Raetz L., Dahl R.H., Tai A., Mahatdejkul-Meadows T., Xu L. (2016). Rewriting yeast central carbon metabolism for industrial isoprenoid production. Nature.

[108] Guadalupe-Medina V., Wisselink H.W., Luttik M.A.H., de Hulster E., Daran J.-M., Pronk J.T., van Maris A.J.A. (2013). Carbon dioxide fixation by Calvin-Cycle enzymes improves ethanol yield in yeast. Biotechnol Biofuels.

[109] Hernandez J.M., Baker S.H., Lorbach S.C., Shively S.M., Tabita F.R. (1996). Deduced amino acid sequence, functional expression, and unique enzymatic properties of the form I and form II ribulose bisphosphate carboxylase/oxygenase from the chemoautotrophic bacterium Thiobacillus denitrificans. J Bacteriol.

[110] Hudson G., Morell M., Arvidsson Y., Andrews T. (1992). Synthesis of spinach phosphoribulokinase and ribulose-1,5-bisphosphate in Escherichia coli. Funct Plant Biol.

[111] Brandes H.K., Hartman F.C., Lu T.-Y.S., Larimer F.W. (1996). Efficient expression of the gene for spinach phosphoribulokinase in *Pichia pastoris* and utilization of the recombinant enzyme to explore the role of regulatory cysteinyl residues by site-directed mutagenesis. J Biol Chem.

[112] Kötter P., Ciriacy M. (1993). Xylose fermentation by Saccharomyces cerevisiae. Appl Microbiol Biotechnol.

[113] Roca C., Nielsen J., Olsson L. (2003). Metabolic engineering of ammonium assimilation in xylose-fermenting *Saccharomyces cerevisiae* improves ethanol production. Appl Environ Microbiol.

[114] Li Y.-J., Wang M.-M., Chen Y.-W., Wang M., Fan L.-H., Tan T.-W. (2017). Engineered yeast with a CO_2_-fixation pathway to improve the bio-ethanol production from xylose-mixed sugars. Sci Rep.

[115] Xia P.-F., Zhang G.-C., Walker B., Seo S.-O., Kwak S., Liu J.-J., Kim H., Ort D.R., Wang S.-G., Jin Y.-S. (2017). Recycling carbon dioxide during xylose fermentation by engineered Saccharomyces cerevisiae. ACS Synth Biol.

[116] Sonderegger M., Schümperli M., Sauer U. (2004). Metabolic engineering of a phosphoketolase pathway for pentose catabolism in Saccharomyces cerevisiae. Appl Environ Microbiol.

[117] Daran-Lapujade P., Jansen M.L.A., Daran J.-M., van Gulik W.M., de Winde J.H., Pronk J.T. (2004). Role of transcriptional regulation in controlling fluxes in central carbon metabolism of Saccharomyces cerevisiae. J Biol Chem.

[118] Pirt S.J. (1965). The maintenance energy of bacteria in growing cultures. Proc R Soc Lond Ser B.

[119] Sánchez B.J., Zhang C., Nilsson A., Lahtvee P.-J., Kerkhoven E.J., Nielsen J. (2017). Improving the phenotype predictions of a yeast genome-scale metabolic model by incorporating enzymatic constraints. Mol Syst Biol.

[120] Regueira A., Lema J.M., Mauricio-Iglesias M. (2021). Microbial inefficient substrate use through the perspective of resource allocation models. Curr Opin Biotechnol.

[121] Hennaut C., Hilger F., Grenson M. (1970). Space limitation for permease insertion in the cytoplasmic membrane of Saccharomyces cerevisiae. Biochem Biophys Res Commun.

[122] Gopal C.V., Broad D., Lloyd D. (1989). Bioenergetic consequences of protein overexpression in Saccharomyces cerevisiae. Appl Microbiol Biotechnol.

[123] Glick B.R. (1995). Metabolic load and heterologous gene expression. Biotechnol Adv.

[124] Mans R., van Rossum H.M., Wijsman M., Backx A., Kuijpers N., van den Broek M., Daran-Lapujade P., Pronk J.T., van Maris A.J.A., Daran J.-M.G. (2015). CRISPR/Cas9: a molecular Swiss army knife for simultaneous introduction of multiple genetic modifications in Saccharomyces cerevisiae. FEMS Yeast Res.

[125] Kuijpers N.G., Solis-Escalante D., Bosman L., van den Broek M., Pronk J.T., Daran J.-M., Daran-Lapujade P. (2013). A versatile, efficient strategy for assembly of multi-fragment expression vectors in *Saccharomyces cerevisiae* using 60 bp synthetic recombination sequences. Microb Cell Factories.

[126] Sánchez B.J., Nielsen J. (2015). Genome scale models of yeast: towards standardized evaluation and consistent omic integration. Integr Biol.

[127] Lopes H., Rocha I. (2017). Genome-scale modeling of yeast: chronology, applications and critical perspectives. FEMS Yeast Res.

[128] Sandberg T.E., Salazar M.J., Weng L.L., Palsson B.O., Feist A.M. (2019). The emergence of adaptive laboratory evolution as an efficient tool for biological discovery and industrial biotechnology. Metab Eng.

[129] Mans R., Daran J.-M.G., Pronk J.T. (2018). Under pressure: evolutionary engineering of yeast strains for improved performance in fuels and chemicals production. Curr Opin Biotechnol.

[130] Radecka D., Mukherjee V., Quintilla Mateo R., Stojiljkovic M., Foulquié-Moreno M.R., Thevelein J.M. (2015). Looking beyond *Saccharomyces*: the potential of non-conventional yeast species for desirable traits in bioethanol fermentation. FEMS Yeast Res.

[131] Della-Bianca B.E., Gombert A.K. (2013). Stress tolerance and growth physiology of yeast strains from the Brazilian fuel ethanol industry. Antonie Leeuwenhoek.

[132] Lane I. (2015 Mar 9). Lallemand acquires patent for pathways which reduce glycerol production. Biofuels Digest.

[133] Lane J. (2018 Jun 14). The battle for yeast supremacy: DSM, DuPont, novozymes and more. Biofuels Digest.

[134] Bryan T. (2020 Dec 28). DuPont nutrition & biosciences - when 'best-of-both' traits shine. Ethanol Prod Mag.

[135] Basso L.C., Basso T.O., Rocha S.N., Dos Santor Bernades M.A. (2011). Biofuel production-recent developments and prospects.

[136] den Haan R., Rose S.H., Cripwell R.A., Trollope K.M., Myburgh M.W., Viljoen-Bloom M., van Zyl W.H. (2021). Heterologous production of cellulose- and starch-degrading hydrolases to expand *Saccharomyces cerevisiae* substrate utilization: lessons learnt. Biotechnol Adv.

[137] Hahn-Hägerdal B., Karhumaa K., Jeppsson M., Gorwa-Grauslund M.F. (2007). Metabolic engineering for pentose utilization in Saccharomyces cerevisiae. Adv Biochem Eng Biotechnol.

[138] Parisutham V., Chandran S.-P., Mukhopadhyay A., Lee S.K., Keasling J.D. (2017). Intracellular cellobiose metabolism and its applications in lignocellulose-based biorefineries. Bioresour Technol.

[139] Ingledew W.M., Walker G.M. (2017). The alcohol textbook.

[140] Gomes D., Cruz M., de Resende M., Ribeiro E., Teixeira J., Domingues L. (2021). Very high gravity bioethanol revisited: main challenges and advances. Fermentation.

[141] Guadalupe‐Medina V., Metz B., Oud B., van Der Graaf C.M., Mans R., Pronk J.T., van Maris A.J. (2014). Evolutionary engineering of a glycerol‐3‐phosphate dehydrogenase‐negative, acetate‐reducing S accharomyces cerevisiae strain enables anaerobic growth at high glucose concentrations. Microb Biotechnol.

[142] Perli T., Wronska A.K., Ortiz-Merino R.A., Pronk J.T., Daran J.M. (2020). Vitamin requirements and biosynthesis in Saccharomyces cerevisiae. Yeast.

[143] Wronska A.K., van den Broek M., Perli T., de Hulster E., Pronk J.T., Daran J.M. (2021). Engineering oxygen-independent biotin biosynthesis in Saccharomyces cerevisiae. Metab Eng.

[144] Wiersma S.J., Mooiman C., Giera M., Pronk J.T. (2020). Squalene-tetrahymanol cyclase expression enables sterol independent growth of Saccharomyces cerevisiae. Appl Environ Microbiol.

[145] Perli T., Vos A.M., Bouwknegt J., Dekker W.J.C., Wiersma S.J., Mooiman C., Ortiz-Merino R.A., Daran J.-M., Pronk J.T. (2021). Identification of oxygen-independent pathways for pyridine nucleotide and coenzyme A synthesis in anaerobic fungi by expression of candidate genes in yeast. mBio.

[146] Lewis S. (2016 Nov 10). Options expand for effective bacterial control in ethanol production. Ethanol Prod Mag.

[147] Kurylenko O.O., Ruchala J., Hryniv O.B., Abbas C.A., Dmytruk K.V., Sibirny A.A. (2014). Metabolic engineering and classical selection of the methylotrophic thermotolerant yeast *Hansenula polymorpha* for improvement of high-temperature xylose alcoholic fermentation. Microb Cell Factories.

[148] Dekker W.J.C., Ortiz-Merino R.A., Kaljouw A., Battjes J., Wiering F.W., Mooiman C., de la Torre Cortés P., Pronk J.T. (2021). Engineering the thermotolerant industrial yeast *Kluyveromyces marxianus* for anaerobic growth. Metab Eng.

