# Supplementary material for: Pathway engineering strategies for improved product yield in yeast-based industrial ethanol production
Source: Synth Syst Biotechnol. 2022 Jan 22;7(1):554–66. doi: 10.1016/j.synbio.2021.12.010 (PMC8792080; doi:10.1016/j.synbio.2021.12.010)
Supplement: Multimedia component 1 [file mmc1.docx]

**Pathway engineering strategies for improved product yield in yeast-based industrial ethanol production**

Aafke C. A. van Aalst^1,#^, Sophie C. de Valk^1,#^, Walter M. van Gulik^1^, Mickel L. A. Jansen^2^, Jack T. Pronk^1^ and Robert Mans^1*^

**Supplementary materials**

**Supplementary Table 1:** Estimated impacts on the biomass-mass specific rate of hexose consumption for different pathway-engineering strategies to improve ethanol yields (see **Table 2** for estimated maximum impacts on ethanol yield), calculated with a stoichiometric model of the core metabolic network of S. cerevisiae [1]. For the three strategies focused on NADH reoxidation, glycerol production was set at zero and oxidation of surplus NADH from biosynthetic reactions was entirely routed through the engineered pathways. Subscript x denotes yeast biomass.

| Specific growth  rate (h^-1^) | -q_hexose_ (mmol/Cmol_x_/h) | | | | | |
| --- | --- | --- | --- | --- | --- | --- |
|  | Reference | Altered ATP coupling of sugar dissimilation | | Alternative pathways for  reoxidation of NADH | | |
|  | Wild type | H^+^ symport/ intracellular hydrolysis of sucrose (yields 1.5 ATP/hexose) | H^+^ symport of glucose  (yields 1 ATP/glucose) | PFL/A-ALD | PK/PTA/A-ALD | PRK/Rubisco |
| 0.3 | 402 | 536 (33.3%) | 803 (100%) | 307 (-23.6%) | 278 (-30.8%) | 308 (-23.4%) |
| 0.1 | 143 | 190 (33.3%) | 285 (100%) | 111 (-22.1%) | 102 (-28.9%) | 111 (-22.0%) |
| 0.03 | 52.1 | 69.4 (33.3%) | 104 (100%) | 42.6 (-18.2%) | 39.7 (-23.8%) | 42.7 (-18.1%) |
| 0.01 | 26.2 | 34.9 (33.3%) | 52.3 (100%) | 23.0 (-12.1%) | 22.0 (-15.8%) | 23.0 (-12.0%) |
| 0.001 | 14.5 | 19.3 (33.3%) | 29.0 (100%) | 14.2 (-2.2%) | 14.1 (-2.8%) | 14.2 (-2.2%) |

**Supplementary Table 2:** Estimated impacts on the biomass yield on hexose units for different pathway-engineering strategies to improve ethanol yields (see Table 1 for estimated maximum impacts on ethanol yield), calculated with a stoichiometric model of the core metabolic network of S. cerevisiae [1]. For the three strategies focused on NADH reoxidation, glycerol production was set at zero and oxidation of surplus NADH from biosynthetic reactions was entirely routed through the engineered pathways.

| Specific growth  rate (h^-1^) | Y_x/hexose_ (g_x_/g) | | | | | |
| --- | --- | --- | --- | --- | --- | --- |
|  | Reference | Altered ATP coupling of sugar dissimilation | | Alternative pathways for  reoxidation of NADH | | |
|  | Wild type | H^+^ symport/ intracellular hydrolysis of sucrose (yields 1.5 ATP/hexose) | H^+^ symport of glucose  (yields 1 ATP/glucose) | PFL/A-ALD | PK/PTA/A-ALD | PRK/Rubisco |
| 0.3 | 0.109 | 0.082 (-25%) | 0.055 (-50%) | 0.143 (30.9%) | 0.158 (44.5%) | 0.143 (30.6%) |
| 0.1 | 0.103 | 0.077 (-25%) | 0.051 (-50%) | 0.132 (28.4%) | 0.145 (40.7%) | 0.132 (28.2%) |
| 0.03 | 0.085 | 0.063 (-25%) | 0.042 (-50%) | 0.103 (22.3%) | 0.111 (31.2%) | 0.103 (22.1%) |
| 0.01 | 0.056 | 0.042 (-25%) | 0.028 (-50%) | 0.064 (13.7%) | 0.067 (18.7%) | 0.064 (13.6%) |
| 0.001 | 0.010 | 0.008 (-25%) | 0.005 (-50%) | 0.010 (2.2%) | 0.010 (2.9%) | 0.010 (2.2%) |

**Model-based estimation of the impact of pathway engineering strategies on growth stoichiometry**

Quantitative estimates of the impact of different pathway engineering strategies on ethanol yield, biomass-specific rate of glucose consumption and biomass yield in anaerobic, glucose-grown cultures were generated with a compartmented stoichiometric network model for growth of *Saccharomyces cerevisiae* [1], with the following modifications:

- Calculations were based on a growth-rate independent rate of ATP turnover for cellular maintenance (m_ATP_) of 1 mmol (g biomass)^-1^ h^-1^ [2] and a growth-coupled ATP cost of 0.5 mol (Cmol biomass)^-1^.
- Molecular and elemental composition of *S. cerevisiae* biomass was assumed to correspond to the statistically reconciled composition calculated by Lange and Heijnen (2001) [3] for glucose-limited cultures grown at a dilution rate of 0.10 h^-1^ (1 Cmol biomass = 26.4 g).
- Pathway engineering strategies were simulated by introducing the following modifications:

H^+^ symport and subsequent intracellular hydrolysis of sucrose

- This scenario was simulated by coupling import of glucose, which in the reference model occurs via facilitated diffusion (no coupling with proton translocation), to the inward translocation of 0.5 H^+^

H^+^ symport of glucose

- This scenario was simulated by coupling import of glucose, which in the reference model occurs via facilitated diffusion (no coupling with proton translocation), to the inward translocation of 1.0 H^+^

Expression of pyruvate-formate lyase (PFL) and acetylating acetaldehyde dehydrogenase (A-ALD)

To simulate this scenario, the following changes were introduced in the model:

- the rate of glycerol production (q_glycerol_) was set to 0
- NAD^+^-dependent acetaldehyde dehydrogenase was removed from the model
- PFL was added to the model: pyruvate 🡪 formate + acetyl-CoA
- A-ALD was added to the model: acetyl-CoA + NADH 🡪 acetaldehyde + NAD^+^
- formate export was assumed to occur by uniport of the formate anion

Expression of phosphoketolase (PK), phosphotransacetylase (PTA) and acetylating acetaldehyde dehydrogenase (A-ALD):

- the rate of glycerol production (q_glycerol_) was set to 0
- NAD^+^-dependent acetaldehyde dehydrogenase (cACTAL deh(NAD)) was removed from the model
- PK was added to the model: xylulose-5P + P_i_ 🡪 glyceraldehyde-3P + acetyl-P
- Phosphotransacetylase was added to the model: acetyl-P + CoA 🡪 acetyl-CoA + P_i_
- A-ALD was added to the model: acetyl-CoA + NADH 🡪 acetaldehyde + NAD^+^

Expression of phosphoribulokinase (PRK) and ribulose-1,5-bisphosphate carboxylase/oxygenase (Rubisco):

- the rate of glycerol production (q_glycerol_) was set at 0
- PRK was added to the model: ribulose-5P + ATP 🡪 ribulose-1,5P + ADP
- Rubisco was added to the model: ribulose-1,5P + CO_2_ 🡪 2 3P-glycerate

The reaction list for stoichiometric model is given below.

amino acid synthesis

alanine transaminase; cALA N-trans

1 GLM:cyt + 1 PYR:cyt <=> 1 ALA:cyt + 1 OGL:cyt

arginine synthesis; cARG syn

1 ASP:cyt + 1 ATP:cyt + 1 CARP:cyt + 1 ORN:cyt <=> 1 AMP:cyt + 1 ARG:cyt + 1 FUM:cyt + 3 H:cyt + 1 Pi:cyt + 1 PPi:cyt

asparagine synthesis; cASN syn

1 ASP:cyt + 1 ATP:cyt + 1 GLN:cyt + 1 H2O:cyt <=> 1 ADP:cyt + 1 ASN:cyt + 1 GLM:cyt + 1 H:cyt + 1 Pi:cyt

asparatate aminotransferase; cASP N-trans

1 GLM:cyt + 1 OXACT:cyt <=> 1 ASP:cyt + 1 OGL:cyt

aspartate kinase; cASP kin

1 ASP:cyt + 1 ATP:cyt + 2 H:cyt + 2 NADPH:cyt <=> 1 ADP:cyt + 1 HSER:cyt + 2 NADP:cyt + 1 Pi:cyt

branched chain amino acid transferase (isoleucine) mit; mILE N-trans

1 GLM:mit + 1 H:mit + 1 NADPH:mit + 1 OBU:mit + 1 PYR:mit <=> 1 CO2:mit + 1 H2O:mit + 1 ILE:mit + 1 NADP:mit + 1 OGL:mit

branched chain amino acid transferase (leucine); cLEU N-trans

1 GLM:cyt + 1 IPM:cyt + 1 NAD:cyt <=> 1 CO2:cyt + 1 LEU:cyt + 1 NADH:cyt + 1 OGL:cyt

branched chain amino acid transferase (valine) cyt; cVAL N-trans

1 GLM:cyt + 1 OIV:cyt => 1 OGL:cyt + 1 VAL:cyt

branched chain amino acid transferase (valine) mit; mVAL N-trans

1 GLM:mit + 1 OIV:mit <=> 1 OGL:mit + 1 VAL:mit

carbamoyl phoshate synthase; cCARP syn

2 ATP:cyt + 1 CO2:cyt + 1 GLN:cyt + 2 H2O:cyt <=> 2 ADP:cyt + 1 CARP:cyt + 1 GLM:cyt + 3 H:cyt + 1 Pi:cyt

cysteine synthese; cCYS syn

2 H:cyt + 1 HCYS:cyt + 1 SER:cyt <=> 1 CYS:cyt + 1 NH4:cyt + 1 OBU:cyt

glutamate ammonia ligase; cGLM N-lig

1 ATP:cyt + 1 GLM:cyt + 1 NH4:cyt <=> 1 ADP:cyt + 1 GLN:cyt + 1 H:cyt + 1 Pi:cyt

glutamate dehydrogenase; cGLM deh

1 H:cyt + 1 NADPH:cyt + 1 NH4:cyt + 1 OGL:cyt <=> 1 GLM:cyt + 1 H2O:cyt + 1 NADP:cyt

glycine hydroxymethyl transferase; cGLY transf

1 SER:cyt + 1 THF:cyt <=> 1 GLY:cyt + 1 H2O:cyt + 1 METHF:cyt

histidine synthesis; cHIS syn

1 ATP:cyt + 1 GLN:cyt + 3 H2O:cyt + 2 NAD:cyt + 1 PRPP:cyt <=> 1 AICAR:cyt + 6 H:cyt + 1 HIS:cyt + 2 NADH:cyt + 1 OGL:cyt + 1 Pi:cyt + 2 PPi:cyt

homocitrate synthesis; cHCIT syn

1 ACCoA:cyt + 1 H2O:cyt + 1 OGL:cyt <=> 1 CoA:cyt + 1 H:cyt + 1 HCIT:cyt

homocysteine synthesis; cHCYS syn

1 ACCoA:cyt + 1 H2S:cyt + 1 HSER:cyt <=> 1 ACT:cyt + 1 CoA:cyt + 2 H:cyt + 1 HCYS:cyt

isopropylmalate synthase cyt; cIPM syn

1 ACCoA:cyt + 1 H2O:cyt + 1 OIV:cyt => 1 CoA:cyt + 1 H:cyt + 1 IPM:cyt

methionine synthase; cMET syn

1 H:cyt + 1 HCYS:cyt + 1 MYTHF:cyt <=> 1 MET:cyt + 1 THF:cyt

ornithine synthesis; mORN syn

1 ATP:mit + 2 GLM:mit + 1 H:mit + 1 NADPH:mit => 1 ADP:mit + 1 NADP:mit + 1 OGL:mit + 1 ORN:mit + 1 Pi:mit

oxoadipate synthesis; mOAD syn

1 HCIT:mit + 1 NAD:mit <=> 1 CO2:mit + 1 NADH:mit + 1 OAD:mit

oxoisovalerate synthesis mit; mOIV syn

2 H:mit + 1 NADPH:mit + 2 PYR:mit <=> 1 CO2:mit + 1 H2O:mit + 1 NADP:mit + 1 OIV:mit

phenylalanine synthesis; cPHE syn

1 CHO:cyt + 1 GLM:cyt + 1 H:cyt <=> 1 CO2:cyt + 1 H2O:cyt + 1 OGL:cyt + 1 PHE:cyt

proline dehydrogenase; cPRO deh

1 ATP:cyt + 1 GLM:cyt + 2 H:cyt + 2 NADPH:cyt <=> 1 ADP:cyt + 1 H2O:cyt + 2 NADP:cyt + 1 Pi:cyt + 1 PRO:cyt

serine synthesis; cSER syn

1 3PG:cyt + 1 GLM:cyt + 1 H2O:cyt + 1 NAD:cyt <=> 1 H:cyt + 1 NADH:cyt + 1 OGL:cyt + 1 Pi:cyt + 1 SER:cyt

shikimate pathway; cSHI path

1 ATP:cyt + 1 E4P:cyt + 1 NADPH:cyt + 2 PEP:cyt <=> 1 ADP:cyt + 1 CHO:cyt + 1 NADP:cyt + 4 Pi:cyt

threonine aldolase; cTHR ald

1 THR:cyt => 1 ACTAL:cyt + 1 GLY:cyt

threonine dehydratase mit; mTHR deh

1 H:mit + 1 THR:mit => 1 NH4:mit + 1 OBU:mit

threonine synthesis; cTHR syn

1 ATP:cyt + 1 H2O:cyt + 1 HSER:cyt <=> 1 ADP:cyt + 1 H:cyt + 1 Pi:cyt + 1 THR:cyt

tryptophan synthesis; cTRP syn

1 CHO:cyt + 1 GLN:cyt + 1 PRPP:cyt + 1 SER:cyt <=> 1 CO2:cyt + 1 GAP:cyt + 1 GLM:cyt + 1 H:cyt + 1 H2O:cyt + 2 Pi:cyt + 1 PYR:cyt + 1 TRP:cyt

tyrosine synthesis; cTYR syn

1 CHO:cyt + 1 GLM:cyt + 1 NADP:cyt <=> 1 CO2:cyt + 1 NADPH:cyt + 1 OGL:cyt + 1 TYR:cyt

biomass formation

biomass formation G4; biom-G4

0.398 CARBHYD:cyt + 0.0037 DNA:cyt + 0.0561 H2O:cyt + 0.101 LIPID:cyt + 0.018 metal:cyt + 0.00245 Pi:cyt + 0.451 PROT:cyt + 0.047 RNA:cyt + 0.00094 SO4:cyt + 0.5 ATP:cyt => 1 biom-G4:ext + 0.5 ADP:cyt + 1 H:cyt + 1 Pi:cyt

C-1 metabolism

dihydrofolate reductase; cDHF red

1 DHF:cyt + 1 H:cyt + 1 NADPH:cyt => 1 NADP:cyt + 1 THF:cyt

methylenetetrahydrofolate dehydrogenase; cMETHF deh

1 FTHF:cyt + 1 H:cyt + 1 NADPH:cyt => 1 H2O:cyt + 1 METHF:cyt + 1 NADP:cyt

methylenetetrahydrofolate reductase; cMETHF red

1 H:cyt + 1 METHF:cyt + 1 NADPH:cyt => 1 MYTHF:cyt + 1 NADP:cyt

phosphoribulokinase; PRK

1 ATP:cyt + 3 H:cyt + 1 RIBU5P:cyt => 1 ADP:cyt + 1 RIBU15P:cyt

ribulose-15-bisphosphate carboxylase oxygenase; Rubisco

1 CO2:cyt + 1 H2O:cyt + 1 RIBU15P:cyt => 2 3PG:cyt + 6 H:cyt

catabolism

acetylating acetaldehyde dehydrogenase; cACACTAL deh(NAD)

1 ACTAL:cyt + 1 CoA:cyt + 1 NAD:cyt <=> 1 ACCoA:cyt + 1 H:cyt + 1 NADH:cyt

sulfate assimilation; cSO4 ass

2 ATP:cyt + 3 H:cyt + 4 NADPH:cyt + 1 SO4:cyt => 1 ADP:cyt + 1 AMP:cyt + 2 H2O:cyt + 1 H2S:cyt + 4 NADP:cyt + 1 Pi:cyt + 1 PPi:cyt

diffusion

CO2 diffusion; eCO2<-cCO2

1 CO2:cyt <=> 1 CO2:ext

intracellular carbon dioxide diffusion; cCO2<-mCO2

1 CO2:mit => 1 CO2:cyt

intracellular water diffusion; cH2O<-mH2O

1 H2O:mit => 1 H2O:cyt

water diffusion; eH2O<-cH2O

1 H2O:cyt <=> 1 H2O:ext

glycerol metabolism

glycerol 3-phosphatase; cGOH3P pho

1 GOH3P:cyt + 1 H2O:cyt => 1 GOH:cyt + 1 Pi:cyt

glycolysis, lower

enolase; cEnol

1 2PG:cyt <=> 1 H2O:cyt + 1 PEP:cyt

glyceraldehyde phosphate dehydrogenase; cGAP deh

1 GAP:cyt + 1 NAD:cyt + 1 Pi:cyt <=> 1 13PG:cyt + 1 H:cyt + 1 NADH:cyt

phosphoglycerate kinase; c13PG kin

1 13PG:cyt + 1 ADP:cyt <=> 1 3PG:cyt + 1 ATP:cyt

phosphoglycerate mutase; c3PG mut

1 3PG:cyt <=> 1 2PG:cyt

pyruvate kinase; cPYR kin

1 ADP:cyt + 1 H:cyt + 1 PEP:cyt => 1 ATP:cyt + 1 PYR:cyt

glycolysis, upper

fructosebisphosphate aldolase; cF16P ald

1 F16P:cyt <=> 1 DHAP:cyt + 1 GAP:cyt

glucose 6-phosphate isomerase; cG6P iso

1 G6P:cyt <=> 1 F6P:cyt

hexokinase; cHX kin

1 ATP:cyt + 1 GLUC:cyt => 1 ADP:cyt + 1 G6P:cyt + 1 H:cyt

phosphofructokinase; cPF kin

1 ATP:cyt + 1 F6P:cyt => 1 ADP:cyt + 1 F16P:cyt + 1 H:cyt

triose phophate isomerase; cTP iso

1 DHAP:cyt <=> 1 GAP:cyt

intracellular transport

ADP-ATP antiport cyt/mit; ADPc<->ATPm

1 ADP:cyt + 1 ATP:mit => 1 ADP:mit + 1 ATP:cyt

mit. ammonium carrier protein; NH4m car

1 H:cyt + 1 NH4:mit => 1 H:mit + 1 NH4:cyt

mit. homocitrate carrier; HCITm car

4 H:cyt + 1 HCIT:cyt => 4 H:mit + 1 HCIT:mit

mit. isoleucine carrier; ILEm car

1 H:cyt + 1 ILE:mit => 1 H:mit + 1 ILE:cyt

mit. ornithine carrier; ORNm car

1 H:cyt + 1 ORN:mit => 1 H:mit + 1 ORN:cyt

mit. oxaloacetate exporter; OXACTm ex

1 H:mit + 1 OXACT:mit => 1 H:cyt + 1 OXACT:cyt

mit. oxobutyrate carrier; OBUm car

1 H:cyt + 1 OBU:cyt => 1 H:mit + 1 OBU:mit

mit. oxogluterate/malate carrier; ODC1m car

1 MAL:cyt + 1 OGL:mit => 1 MAL:mit + 1 OGL:cyt

mit. oxogluterate/oxoadipate carrier; ODC2m car

1 OAD:mit + 1 OGL:cyt => 1 OAD:cyt + 1 OGL:mit

mit. oxoisovalerate carrier; OIVm car

1 OIV:mit => 1 OIV:cyt

mit. phosphate carrier mit; Pi_m car

2 H:cyt + 1 Pi:cyt => 2 H:mit + 1 Pi:mit

mit. pyruvate proton symport; PYRm car

2 H:cyt + 1 PYR:cyt => 2 H:mit + 1 PYR:mit

mit. threonine carrier; THRm car

1 H:cyt + 1 THR:cyt => 1 H:mit + 1 THR:mit

mit. valine importer; VALm imp

1 H:cyt + 1 VAL:cyt => 1 H:mit + 1 VAL:mit

NADH shuttle; NADH shuttle

1 NAD:cyt + 1 NADH:mit => 1 NAD:mit + 1 NADH:cyt

succinate malate carrier; mSUC MAL car

1 MAL:cyt + 1 SUC:mit <=> 1 MAL:mit + 1 SUC:cyt

lipid synthesis

acetyl-CoA carboxylase; cACCoA carb

1 ACCoA:cyt + 1 ATP:cyt + 1 CO2:cyt + 1 H2O:cyt => 1 ADP:cyt + 2 H:cyt + 1 MACoA:cyt + 1 Pi:cyt

adenosly homocysteinase; cSAH hyd

1 H2O:cyt + 1 SAH:cyt => 1 A:cyt + 1 H:cyt + 1 HCYS:cyt

average fatty acid formation; cAvFA form

1.7 OLE-CoA:cyt + 4.4 PLLM-CoA:cyt + 1.4 PLM-CoA:cyt + 1 STE-CoA:cyt => 8.5 avFA-CoA:cyt

average phospholipid formation; cAvPL form

11 PHD-CHO:cyt + 4 PHD-ETA:cyt + 3 PHD-SER:cyt => 18 avPL:cyt

FAT formation; cFAT form

1 avFA-CoA:cyt + 1 H2O:cyt + 1 PHD:cyt => 1 CoA:cyt + 1 FAT:cyt + 1 Pi:cyt

glycerol 3-phosphate acyltransferase; cGOH3P trans

2 avFA-CoA:cyt + 1 GOH3P:cyt => 2 CoA:cyt + 1 PHD:cyt

glycerol 3-phosphate dehydrogenase; cGOH3P deh

1 DHAP:cyt + 1 H:cyt + 1 NADH:cyt => 1 GOH3P:cyt + 1 NAD:cyt

methionine adenosyl transferase; cMET Atrans

1 ATP:cyt + 2 H2O:cyt + 1 MET:cyt => 1 H:cyt + 3 Pi:cyt + 1 SAM:cyt

oleate CoA ligase; OLE CoA lig

1 ATP:cyt + 1 CoA:cyt + 1 H2O:cyt + 1 OLE:cyt => 1 AMP:cyt + 1 H:cyt + 1 OLE-CoA:cyt + 2 Pi:cyt

palmitate CoA ligase; cPLM lig

1 ATP:cyt + 1 CoA:cyt + 1 H2O:cyt + 1 PLM:cyt => 1 AMP:cyt + 1 H:cyt + 2 Pi:cyt + 1 PLM-CoA:cyt

palmitic acid synthesis; cPLM syn

1 ACCoA:cyt + 20 H:cyt + 7 MACoA:cyt + 14 NADPH:cyt => 7 CO2:cyt + 8 CoA:cyt + 6 H2O:cyt + 14 NADP:cyt + 1 PLM:cyt

palmitoleate CoA ligase; PLLM CoA lig

1 ATP:cyt + 1 CoA:cyt + 1 H2O:cyt + 1 PLLM:cyt => 1 AMP:cyt + 1 H:cyt + 2 Pi:cyt + 1 PLLM-CoA:cyt

phosphatidate cytidyl transferase; cPHD-C trans

1 CTP:cyt + 1 H2O:cyt + 1 PHD:cyt => 1 CMP-DGOH:cyt + 2 Pi:cyt

phosphatidyl-ethanolamine methyltransferase; cPHD-EA mtrans

1 PHD-ETA:cyt + 3 SAM:cyt => 3 H:cyt + 1 PHD-CHO:cyt + 3 SAH:cyt

phosphatidyl-serine decarboxylase; cPHD-SER dcarb

1 PHD-SER:cyt => 1 CO2:cyt + 1 PHD-ETA:cyt

phosphatidyl-serine synthase; cPHD-SER syn

1 CMP-DGOH:cyt + 1 SER:cyt => 1 CMP:cyt + 1 PHD-SER:cyt

stearate CoA ligase; cSTE ligase

1 ATP:cyt + 1 CoA:cyt + 1 H2O:cyt + 1 STE:cyt => 1 AMP:cyt + 1 H:cyt + 2 Pi:cyt + 1 STE-CoA:cyt

stearic acid synthesis; cSTE syn

1 ACCoA:cyt + 23 H:cyt + 8 MACoA:cyt + 16 NADPH:cyt => 8 CO2:cyt + 9 CoA:cyt + 7 H2O:cyt + 16 NADP:cyt + 1 STE:cyt

macromolecule synthesis

average amino acid formation; cAvAA form

0.977 ALA:cyt + 0.386 ARG:cyt + 0.408 ASN:cyt + 0.52 ASP:cyt + 0.0139 CYS:cyt + 1.02 GLM:cyt + 0.526 GLN:cyt + 0.889 GLY:cyt + 0.193 HIS:cyt + 0.589 ILE:cyt + 0.801 LEU:cyt + 0.657 LYS:cyt + 0.114 MET:cyt + 0.0238 ORN:cyt + 0.376 PHE:cyt + 0.422 PRO:cyt + 0.533 SER:cyt + 0.557 THR:cyt + 0.0649 TRP:cyt + 0.196 TYR:cyt + 0.733 VAL:cyt <=> 10 avAA:cyt

carbohydrate synthesis; cCARBHYD syn

1 ATP:cyt + 1 G6P:cyt + 1 H2O:cyt => 1 ADP:cyt + 6 CARBHYD:cyt + 1 H:cyt + 2 Pi:cyt

DNA polymerisation; cDNA poly

0.3 ATP:cyt + 0.2 CTP:cyt + 0.2 GTP:cyt + 1 H:cyt + 0.3 METHF:cyt + 1 NADPH:cyt + 0.3 UTP:cyt => 0.3 DHF:cyt + 9.8 DNA:cyt + 1 H2O:cyt + 1 NADP:cyt + 1 PPi:cyt

lipid formation; cLipid form

0.45 avPL:cyt + 0.55 FAT:cyt => 47.2 LIPID:cyt

lysine synthesis; cLYS syn

1 ATP:cyt + 2 GLM:cyt + 1 NAD:cyt + 2 NADPH:cyt + 1 OAD:cyt <=> 1 AMP:cyt + 1 LYS:cyt + 1 NADH:cyt + 2 NADP:cyt + 2 OGL:cyt + 1 PPi:cyt

protein polymerisation; cPROT poly

3 ATP:cyt + 1 avAA:cyt + 2 H2O:cyt <=> 2 ADP:cyt + 1 AMP:cyt + 4 H:cyt + 2 Pi:cyt + 1 PPi:cyt + 4.81 PROT:cyt

RNA polymerisation; cRNA syn

0.3 ATP:cyt + 0.2 CTP:cyt + 0.2 GTP:cyt + 0.3 UTP:cyt => 1 PPi:cyt + 9.5 RNA:cyt

maintenance

maintenance; cMaintenance

1 ATP:cyt + 1 H2O:cyt => 1 ADP:cyt + 1 H:cyt + 1 Pi:cyt

nucleotide synthesis

adenosine kinase; cA kin

1 A:cyt + 1 ATP:cyt <=> 1 ADP:cyt + 1 AMP:cyt + 1 H:cyt

adenylate kinase; cAMP kin

1 AMP:cyt + 1 ATP:cyt <=> 2 ADP:cyt

AMP synthesis; cAMP syn

1 ASP:cyt + 1 ATP:cyt + 1 IMP:cyt <=> 1 ADP:cyt + 1 AMP:cyt + 1 FUM:cyt + 2 H:cyt + 1 Pi:cyt

CTP synthetase; cCTP syn

1 ATP:cyt + 1 GLN:cyt + 1 H2O:cyt + 1 UTP:cyt <=> 1 ADP:cyt + 1 CTP:cyt + 1 GLM:cyt + 2 H:cyt + 1 Pi:cyt

cytidylate kinase; cCMP kin

1 ATP:cyt + 1 CMP:cyt <=> 1 ADP:cyt + 1 CDP:cyt

GMP synthesis; cGMP syn

1 ATP:cyt + 1 GLN:cyt + 2 H2O:cyt + 1 IMP:cyt + 1 NAD:cyt <=> 1 AMP:cyt + 1 GLM:cyt + 1 GMP:cyt + 4 H:cyt + 1 NADH:cyt + 1 PPi:cyt

guanylate kinase; cGMP kin

1 ATP:cyt + 1 GMP:cyt => 1 ADP:cyt + 1 GDP:cyt

IMP synthesis; cIMP syn

1 AICAR:cyt + 1 FTHF:cyt => 1 H2O:cyt + 1 IMP:cyt + 1 THF:cyt

nucleoside diphosphate kinase 1; cGDP kin

1 ATP:cyt + 1 GDP:cyt <=> 1 ADP:cyt + 1 GTP:cyt

nucleoside diphosphate kinase 2; cUDP kin

1 ATP:cyt + 1 UDP:cyt => 1 ADP:cyt + 1 UTP:cyt

nucleoside diphosphate kinase 3; cCDP kin

1 ATP:cyt + 1 CDP:cyt => 1 ADP:cyt + 1 CTP:cyt

phosphoribosyl pyrophosphate synthesis; cPRPP syn

1 ATP:cyt + 1 RIBU5P:cyt <=> 1 AMP:cyt + 1 H:cyt + 1 PRPP:cyt

phosphoribosyl-5-amino 4-imidazole carboxamide; cAICAR syn

1 ASP:cyt + 4 ATP:cyt + 1 CO2:cyt + 1 FTHF:cyt + 2 GLN:cyt + 1 GLY:cyt + 2 H2O:cyt + 1 PRPP:cyt <=> 4 ADP:cyt + 1 AICAR:cyt + 1 FUM:cyt + 2 GLM:cyt + 8 H:cyt + 4 Pi:cyt + 1 PPi:cyt + 1 THF:cyt

pyrophosphatase; cPPi ase

1 H2O:cyt + 1 PPi:cyt => 2 Pi:cyt

UMP synthesis anaerobic; cUMP syn an

1 ASP:cyt + 1 CARP:cyt + 1 NAD:cyt + 1 PRPP:cyt <=> 1 CO2:cyt + 1 H:cyt + 1 H2O:cyt + 1 NADH:cyt + 1 Pi:cyt + 1 PPi:cyt + 1 UMP:cyt

uridylate kinase; cUMP kin

1 ATP:cyt + 1 UMP:cyt <=> 1 ADP:cyt + 1 UDP:cyt

Mitochondrial ATPase

F1-F0 ATPase; mATPase

3 ADP:mit + 10 H:cyt + 3 Pi:mit <=> 3 ATP:mit + 7 H:mit + 3 H2O:mit

pentose phosphate pathway

glucose-6-phosphate dehydrogenase (NADP); cG6P deh

1 G6P:cyt + 1 H2O:cyt + 2 NADP:cyt => 1 CO2:cyt + 2 H:cyt + 2 NADPH:cyt + 1 RIBU5P:cyt

ribosephosphate isomerase; cRIBU iso

1 RIBU5P:cyt <=> 1 RIB5P:cyt

ribulosephosphate 3-epimerase; cRIBUP epi

1 RIBU5P:cyt <=> 1 XYL5P:cyt

transaldolase; cTA1

1 GAP:cyt + 1 SED7P:cyt <=> 1 E4P:cyt + 1 F6P:cyt

transketolase 1; cTK1

1 RIB5P:cyt + 1 XYL5P:cyt <=> 1 GAP:cyt + 1 SED7P:cyt

transketolase 2; cTK2

1 E4P:cyt + 1 XYL5P:cyt <=> 1 F6P:cyt + 1 GAP:cyt

pyruvate branchpoint

acetaldehyde dehydrogenase (NAD); cACTAL deh (NAD)

1 ACTAL:cyt + 1 H2O:cyt + 1 NAD:cyt <=> 1 ACT:cyt + 2 H:cyt + 1 NADH:cyt

acetylating NAD-dependent acetaldehyde dehydrogenase; cACTAL deh (acetylating)

1 ACTAL:cyt + 1 CoA:cyt + 1 NAD:cyt <=> 1 ACCoA:cyt + 1 H:cyt + 1 NADH:cyt

acetyl-CoA synthase; cACCoA syn

1 ACT:cyt + 1 ATP:cyt + 1 CoA:cyt + 1 H2O:cyt => 1 ACCoA:cyt + 1 AMP:cyt + 1 H:cyt + 2 Pi:cyt

alcohol dehydrogenase; cETOH deh

1 ACTAL:cyt + 1 H:cyt + 1 NADH:cyt <=> 1 ETOH:cyt + 1 NAD:cyt

lactate dehydrogenase; cLAC deh

1 LAC:cyt + 1 NAD:cyt => 1 H:cyt + 1 NADH:cyt + 1 PYR:cyt

pyruvate carboxylase; cPYR carb

1 ATP:cyt + 1 CO2:cyt + 1 H2O:cyt + 1 PYR:cyt => 1 ADP:cyt + 2 H:cyt + 1 OXACT:cyt + 1 Pi:cyt

pyruvate decarboxylase; cPYR dec

1 H:cyt + 1 PYR:cyt => 1 ACTAL:cyt + 1 CO2:cyt

pyruvate dehydrogenase mit; mPYR deh

1 CoA:mit + 1 NAD:mit + 1 PYR:mit => 1 ACCoA:mit + 1 CO2:mit + 1 NADH:mit

Pyruvate formate lyase; PFL

1 CoA:cyt + 1 PYR:cyt => 1 ACCoA:cyt + 1 FOR:cyt

TCA cycle

aconitase 1 mit; mACON 1

1 CIT:mit => 1 ACO:mit + 1 H2O:mit

aconitase 2 mit; mACON 2

1 ACO:mit + 1 H2O:mit => 1 ICIT:mit

citrate synthase mit; mCIT syn

1 ACCoA:mit + 1 H2O:mit + 1 OXACT:mit => 1 CIT:mit + 1 CoA:mit + 1 H:mit

fumarate hydrase; cFUM hy

1 FUM:cyt + 1 H2O:cyt <=> 1 MAL:cyt

fumarate hydrase mit; mFUM hy

1 FUM:mit + 1 H2O:mit <=> 1 MAL:mit

fumarate reductase mit; mFUM red

1 FUM:mit + 1 H:mit + 1 NADH:mit <=> 1 NAD:mit + 1 SUC:mit

isocitrate dehydrogenase (NAD) mit; mICIT deh_NAD

1 ICIT:mit + 1 NAD:mit => 1 CO2:mit + 1 NADH:mit + 1 OGL:mit

isocitrate dehydrogenase (NADP) mit; mICIT deh_NADP

1 ICIT:mit + 1 NADP:mit => 1 CO2:mit + 1 NADPH:mit + 1 OGL:mit

malate dehydrogenase; cMAL deh

1 MAL:cyt + 1 NAD:cyt <=> 1 H:cyt + 1 NADH:cyt + 1 OXACT:cyt

malate dehydrogenase mit; mMAL deh

1 MAL:mit + 1 NAD:mit <=> 1 H:mit + 1 NADH:mit + 1 OXACT:mit

transport

acetate transport; eACT trans

1 ACT:ext + 1 H:ext => 1 ACT:cyt + 1 H:cyt

ATPase plasmamembrane; eATPase

1 ATP:cyt + 1 H2O:cyt => 1 ADP:cyt + 1 H:ext + 1 Pi:cyt

ethanol transport; eETOH trans

1 ETOH:cyt => 1 ETOH:ext

glucose uptake; eGLUC fdiff

1 GLUC:ext => 1 GLUC:cyt

glycerol transport; eGOH trans

1 GOH:cyt + 1 H:ext <=> 1 GOH:ext + 1 H:cyt

lactate excretion; cLAC trans

1 H:cyt + 1 LAC:cyt => 1 H:ext + 1 LAC:ext

malate export; Mal exp

1 H:ext + 1 MAL:cyt => 1 H:cyt + 1 MAL:ext

metal import; eMET imp

1 metal:ext => 1 metal:cyt

NH4 transport; eNH4 trans

1 NH4:ext => 1 NH4:cyt

oleate transport; OLE trans

1 OLE:ext => 1 OLE:cyt

palmitoleate transport; PLLM trans

1 PLLM:ext => 1 PLLM:cyt

phosphate transport; ePi trans

2 H:ext + 1 Pi:ext => 2 H:cyt + 1 Pi:cyt

SO4 transport; eSO4 trans

3 H:ext + 1 SO4:ext => 3 H:cyt + 1 SO4:cyt

succinate transport; suc trans

1 H:ext + 1 SUC:cyt => 1 H:cyt + 1 SUC:ext

Component List

Name ShortName Composition

1,3-phosphoglycerate 13PG C3H4O10P2-4

2-phosphoglycerate 2PG C3H4O7P-3

3-phosphoglycerate 3PG C3H4O7P-3

acetaldehyde ACTAL C2H4O

acetate ACT C2H3O2-1

acetyl-CoA ACCoA C23H34N7O17P3S-4

adenosine A C10H13N5O4

ADP ADP C10H12N5O10P2-3

alanine ALA C3H7NO2

AMP AMP C10H12N5O7P-2

arginine ARG C6H15N4O2+1

asparagine ASN C4H8N2O3

aspartate ASP C4H6NO4-1

ATP ATP C10H12N5O13P3-4

average amino acid avAA C4.81H9.61N1.32O2.53S0.0128-0.0282

AVERAGE FATTY ACID-CoA avFA-CoA C37.6H61.8N7O17P3S-4

average phospholipid avPL C40.3H77.3NO8.33P

biomass G4 biom-G4 CH1.74N0.145O0.595P0.00873S0.0018X0.018

carbamoyl phoshate CARP CH2NO5P-2

CARBOHYDRATES CARBHYD CH1.67O0.833

Carbon dioxide CO2 CO2

CDP CDP C9H12N3O11P2-3

chorismate CHO C10H8O6-2

cis-aconitate ACO C6H3O6-3

citrate CIT C6H5O7-3

citydine diphosphate-diacylglycerol CMP-DGOH C45.3H78.7N3O15P2-2

CMP CMP C9H12N3O8P-2

CoA CoA C21H32N7O16P3S-4

CTP CTP C9H12N3O14P3-4

cystein CYS C3H7NO2S

dihydrofolate DHF C19H19N7O6-2

dihydroxyacetone-phosphate DHAP C3H5O6P-2

DNA DNA CH1.26N0.378O0.612P0.102

E4P E4P C4H7O7P-2

ETOH ETOH C2H6O

FAT FAT C52.9H97.5O6

Formate FOR CHO2-1

formyl-THF FTHF C20H21N7O7-2

fructose 1,6-bisphosphate F16P C6H10O12P2-4

fructose 6-phosphate F6P C6H11O9P-2

fumarate FUM C4H2O4-2

GDP GDP C10H12N5O11P2-3

glucose GLUC C6H12O6

glucose 6-phosphate G6P C6H11O9P-2

glutamate GLM C5H8NO4-1

glutamine GLN C5H10N2O3

glyceraldehyde 3-phosphate GAP C3H5O6P-2

glycerol GOH C3H8O3

glycerol 3-phosphate GOH3P C3H7O6P-2

glycine GLY C2H5NO2

GMP GMP C10H12N5O8P-2

GTP GTP C10H12N5O14P3-4

histidine HIS C6H10N3O2+1

homocitrate HCIT C7H7O7-3

homocysteine HCYS C4H8NO2S-1

homoserine HSER C4H9NO3

hydrogen H H+1

IMP IMP C10H11N4O8P-2

isocitrate ICIT C6H5O7-3

isoleucine ILE C6H13NO2

isopropylmalate IPM C7H10O5-2

lactate LAC C3H5O3-1

leucine LEU C6H13NO2

LIPID LIPID CH1.87N0.00953O0.149P0.00953

lysine LYS C6H15N2O2+1

malate MAL C4H4O5-2

malonyl-CoA MACoA C24H33N7O19P3S-5

metal metal X

metheonine MET C5H11NO2S

methylene-THF METHF C20H21N7O6-2

methyl-THF MYTHF C20H23N7O6-2

NAD NAD +1

NADH NADH H

NADP NADP +1

NADPH NADPH H

NH4 NH4 H4N+1

oleic acid OLE C18H33O2-1

oleoyl-CoA OLE-CoA C39H64N7O17P3S-4

ornithine ORN C5H13N2O2+1

oxaloacetate OXACT C4H2O5-2

oxoadipate OAD C6H6O5-2

oxobutyrate OBU C4H6O3

oxoglutarate OGL C5H4O5-2

oxoisovalerate OIV C5H7O3-1

palmitic acid PLM C16H31O2-1

palmitoleic acid PLLM C16H29O2-1

palmitoleoyl-CoA PLLM-CoA C37H60N7O17P3S-4

palmityl-CoA PLM-CoA C37H62N7O17P3S-4

phenylalanine PHE C9H11NO2

phosphate Pi HO4P-2

phosphatidate PHD C36.3H66.7O8P-2

phosphatidyl-choline PHD-CHO C41.3H79.7NO8P

phosphatidyl-ethanolamine PHD-ETA C38.3H73.7NO8P

phosphatidyl-serine PHD-SER C39.3H73.7NO10P

phosphoenol-pyruvate PEP C3H2O6P-3

phosphoribosyl-formamido-imidazole-carboamide AICAR C9H13N4O8P-2

proline PRO C5H9NO2

PROTEIN PROT CH1.58N0.275O0.318S0.00265-0.00587

PRPP PRPP C5H8O14P3-5

pyrophosphate PPi O7P2-4

pyruvate PYR C3H3O3-1

RIB5P RIB5P C5H9O8P-2

RIBU5P RIBU5P C5H9O8P-2

ribulose-15-bisphosphate RIBU15P C5H12O11P2

RNA RNA CH1.23N0.389O0.737P0.105

s-adenosyl-homocysteine SAH C14H20N6O5S

s-adenosylmetheonine SAM C15H23N6O5S+1

SED7P SED7P C7H13O10P-2

serine SER C3H7NO3

stearate STE C18H35O2-1

stearoyl-CoA STE-CoA C39H66N7O17P3S-4

succinate SUC C4H4O4-2

sulfide H2S H2S

sulphate SO4 O4S-2

tetrahydrofolate THF C19H21N7O6-2

threonine THR C4H9NO3

tryptophane TRP C11H12N2O2

tyrosine TYR C9H11NO3

UDP UDP C9H11N2O12P2-3

UMP UMP C9H11N2O9P-2

UTP UTP C9H11N2O15P3-4

valine VAL C5H11NO2

water H2O H2O

XYL5P XYL5P C5H9O8P-2

**Literature reference**

[1] Daran-Lapujade P, Jansen MLA, Daran J-M, van Gulik WM, de Winde JH, Pronk JT. Role of Transcriptional Regulation in Controlling Fluxes in Central Carbon Metabolism of *Saccharomyces cerevisiae.* The Journal of Biological Chemistry 2004;279(10):9125–9138. <https://doi.org/10.1074/jbc.M309578200>.

[2] Boender LGM, de Hulster E, van Maris AJA, Daran-Lapujade P, Pronk JT. Quantitative Physiology of *Saccharomyces cerevisiae* at Near-Zero Specific Growth Rates*.* Applied and Environmental Microbiology 2009;75(17):5607-5617. <https://doi.org/10.1128/AEM.00429-09>.

[3] Lange HC, Heijnen JJ. Statistical Reconciliation of the Elemental and Molecular Biomass Composition of *Saccharomyces cerevisiae.* Biotechnology and Bioengineering 2001;75(3):334-344. <https://doi.org/10.1002/bit.10054>.
